# Prevalence of asthma among children and adolescents in WHO’s Eastern Mediterranean Region: a meta-analysis of over 0.5 million participants

**DOI:** 10.1186/s12889-024-18716-2

**Published:** 2024-08-07

**Authors:** Mohammad Reza Taherian, Farbod Fatemian, Aram Halimi, Yaser Soleimani, Goljamal Jorjani, Parisa Nozari, Alireza Mosavi Jarrahi, Seyed Saeed Hashemi Nazari, Nabeel Al-Yateem, Amina Al-Marzouqi, Aysha Humid, Syed Azizur Rahman

**Affiliations:** 1 Department of Epidemiology, School of Public Health and Safety, Shahid Beheshti University of Medical Sciences, Tehran, Iran; 2 Medical School, Shahid Beheshti University of Medical Sciences, Tehran, Iran; 3 Department of Nursing, College of Health Sciences, University of Sharjah, Sharjah, UAE; 4 College of Health Sciences, University of Sharjah, Sharjah, UAE; 5 Student Research Committee, School of Public Health and Safety, Shahid Beheshti University of Medical Sciences, Tehran, Iran; 6 Research Center for Social Determinants of Health, Research Institute for Endocrine Sciences, Shahid Beheshti University of Medical Sciences, Tehran, Iran; 7 Prevention of Cardiovascular Disease Research Center, Department of Epidemiology, School of Public Health and Safety, Shahid Beheshti University of Medical Sciences, Tehran, Iran; 8 Safety Promotion and Injury Prevention Research Center, Department of Epidemiology, School of Public Health and Safety, Shahid Beheshti University of Medical Sciences, Tehran, Iran; 9 Department of Health Care Management, College of Health Sciences, University of Sharjah, University of Sharjah, UAE

**Keywords:** Asthma prevalence, Children, Adolescents, EMRO

## Abstract

**Objective:**

This study aims to evaluate the epidemiology of asthma among children and adolescents in the Eastern Mediterranean Region.

**Methods:**

Exhaustive searches were conducted across databases, including PubMed, Scopus, Web of Knowledge Core Collection, Embase, and Google Scholar. The selection criteria included studies reporting asthma prevalence in individuals aged 0 to 19 years, using validated questionnaires. Data were extracted and synthesized using the DerSimonian and Laird random effects model.

**Results:**

The overall prevalence of asthma in Eastern Mediterranean Regional Office (EMRO) countries, among the 514,468 children and adolescents included in this meta-analysis, was 10.61%, synthesized from 95 studies. Among the countries studied, Qatar exhibited the highest prevalence at 16.69%, followed by Saudi Arabia at 16.57%, Iraq at 16.22%, Oman at 15.20%, and Afghanistan at 14.90%. Adolescents showed a slightly higher prevalence of asthma at 10.10% compared to children at 9.70%. Boys exhibited a higher prevalence at 11.48% compared to girls at 9.75%. Urban areas demonstrated a higher prevalence at 11.27% than rural areas at 8.29%.

**Conclusion:**

Efforts to reduce asthma prevalence in Arab countries and address underdiagnosis in African nations within the EMRO are crucial. Targeted interventions should focus on addressing environmental triggers and improving access to healthcare. Enhanced diagnostic capabilities and healthcare infrastructure are necessary in African countries. Collaborative action is essential to alleviate the asthma burden and promote respiratory health across the EMRO region.

**Supplementary Information:**

The online version contains supplementary material available at 10.1186/s12889-024-18716-2.

## Introduction

In recent decades, the prevalence of asthma has increased significantly, becoming a major public health concern, especially among children and teenagers. Asthma, a complex respiratory disorder, is characterized by persistent inflammation of the airways, resulting in recurrent episodes of wheezing, breathlessness, chest tightness, and coughing. These symptoms can significantly impair the quality of life, limit physical activities, and impose a substantial financial burden on healthcare systems and families. While extensive research has explored the prevalence of asthma in different parts of the world, it is evident that several variables contribute significantly to its prevalence [1–4]. Various factors, including age, sex, economic status, genetics, and exposure to pollutants, have been demonstrated to impact the occurrence and severity of asthma [5]. First, age plays a vital role in determining asthma, as it influences the development and duration of the disease. Asthma usually occurs in childhood and the risk decreases as individuals enter adulthood. However, some asthma attacks may begin in old age or later in life, suggesting age-related differences in their prevalence [6]. Gender is another factor affecting asthma. Many studies have shown that men and women have different rates of asthma. Boys have more asthma in childhood, but this trend often changes with more women in adolescence and adulthood [7]. Economic conditions have also been shown to affect asthma. People from lower socioeconomic backgrounds often face challenges such as poor housing, lack of healthcare and environmental risks. These factors lead to higher rates of asthma in low-income communities [8]. Exposure to air pollution is a well-known risk factor for asthma. Breathing in various environmental irritants and pollutants such as smoking, air pollution, allergies, and occupational exposure can cause and lead to asthma. People living in cities or near industrial areas may be particularly susceptible to asthma due to increased levels of air pollution [9].

The Eastern Mediterranean Regional Office (EMRO) of the World Health Organization (WHO), encompassing a vast area and comprising diverse nations, represents a unique intersection of socio-economic, social, and environmental variables that can influence the prevalence and management of asthma among children and teenagers. With nations extending from high-income countries with progressed healthcare frameworks to those hooking with financial challenges and restricted therapeutic assets, the EMRO Locale gives a complicated embroidered artwork for studying the prevalence of asthma [10–12].

The 2013 International Study of Asthma and Allergies in Childhood (ISAAC) Phase Three revealed that the Eastern Mediterranean region had a comparatively lower incidence of asthma symptoms compared to other parts of the world. Specifically, in the 13–14 age group, boys exhibited a prevalence of 10.6%, while girls showed 7.9% for current asthma symptoms [13]. However, despite this lower prevalence, the region still contends with a substantial burden due to the large number of children and adolescents. Previous studies have documented wide variations in asthma prevalence across different countries and areas in the region, ranging from 1.41% to over 20% [14, 15].

Interestingly, there are conflicting findings regarding asthma prevalence in rural areas. For instance, one study suggests a higher prevalence in rural settings than urban ones (20.5% vs. 7.5%), while another indicates lower rates in rural areas compared to urban settings (1.2% vs. 1.9%) [16, 17]. This variation also extends to differences based on gender and location, highlighting a notable research gap.

To address these complexities, this study aims to contribute to our understanding of childhood asthma in the Eastern Mediterranean Region. Our goal is to conduct a thorough review of studies reporting asthma prevalence, providing updated regional and country-specific estimates.

## Methods and materials

This manuscript follows the PRISMA (Preferred Reporting Items for Systematic Reviews and Meta-Analyses) guidelines for clear reporting of the prevalence of asthma among children and adolescents in WHO’s Eastern Mediterranean Region [18]. Our study is registered in the International Prospective Register of Systematic Reviews (PROSPERO: CRD42023379776).

### Search strategy

Data were found using keywords such as asthma, prevalence, epidemiology, children, pediatrics, adolescent, name of the 21 countries located in EMRO including Afghanistan, Bahrain, Djibouti, Egypt, Iran, Iraq, Jordan, Kuwait, Lebanon, Libya, Morocco, Oman, Pakistan, Palestine, Qatar, Saudi Arabia, Somalia, Sudan, Syria, Tunisia, United Arab Emirates, Yemen. Also, the names of big cities of each country were included in the search strategy. We searched PubMed, Scopus, Web of Knowledge Core Collection, Embase, and Google Scholar from their inception until 1 April 2023. Additionally, the search was updated on 23 January to find relevant studies (Appendix A). Finally, citations of the included articles were searched to identify any additional relevant studies.

### Inclusion criteria

Studies that investigated the prevalence of asthma in children under the age of 19 were included in this systematic review without restriction to any specific language. Studies used valid and reliable questionnaires to investigate the prevalence of asthma such as the ISAAC questionnaire, were included. The participants were grouped based on their age, with children defined as those between the ages of 0 and 10, and adolescents classified as those aged 11 to 19. In this study, we aimed to encompass all types of observational studies suitable for assessing asthma prevalence, including cross-sectional, panel studies, and cohorts, while excluding review articles, letters to editors, case reports, case-control studies, and case series.

### Exclusion criteria

Exclusion criteria for this study included unrelated subjects, research conducted outside the EMRO region to assess asthma prevalence, usage of non-standardized questionnaires, incomplete data, and studies that did not specify the age group. Additionally, review articles and case-control studies were also excluded.

### Quality assessment

For quality assessment, we used the Joanna Briggs Institute (JBI) Tool for Prevalence Studies, which contains 9 questions about the sampling frame, sampling method, validity and reliability of the outcome measurement, and statistical analysis of the study. In this checklist, scoring is shown by a scale of “No”, “Yes”, “unclear” and “Not Applicable”. Although there is no reference guide for scoring the checklist questions, we assigned a score of 1 to the “yes” answer, a score of 0.5 to the “unclear” answer, and a score of 0 to the “no” answer for each question. Finally, the Total JBI score was obtained by summing the score obtained from all the answers for a study divided by the total number of questions. Regarding the median JBI score among all studies (0.75) Studies with a total JBI score of 0.75 and higher were considered low-risk studies while studies with a JBI score of less than 0.75 were labeled as high-risk of bias studies. Two reviewers conducted the quality assessment of included studies independently, with any discrepancies resolved through discussion and consensus with a third reviewer.

### Data extraction

The current study made use of the data obtained from included articles via a checklist designed for data extraction. This checklist comprises the author’s name, study design, publication year, the score obtained from JBI quality assessment tool, country, city, sample size, overall age range, gender, asthma prevalence in total, and also in the subgroups of gender, age and place of residence. Data extraction was performed by two reviewers independently, followed by a discussion and consensus with a third reviewer to solve discrepancies. The process of checking studies for inclusion, quality assessment, and data extraction was conducted by FF, YS, PN, and GJ, all of whom were involved in all three processes.

### Statistical analysis

The prevalence of asthma was determined by calculating a pooled estimate using DerSimonian and Laird random effects model [19]. This model was selected due to its ability to accommodate significant heterogeneity arising from variations in assessment methods, demographics, and geographic factors among included studies, thereby providing a more conservative estimate of prevalence. Furthermore, the random effects model acknowledges that the prevalence of asthma in the EMRO follows a distribution, whereas a fixed effect assumes that there is only one true underlying effect size. The results were reported with a 95% confidence interval obtained through an exact method for calculating confidence intervals. To assess heterogeneity, the I2 threshold was applied. Additionally, a subgroup analysis was conducted to examine variations based on geographical areas, age, gender, year of publication, and place of residence. To identify potential reasons for heterogeneity, meta-regression was employed by considering the sample size and publication years. Publication bias was evaluated using Egger’s test and a Doi plot. To evaluate the potential impact of language differences in the included studies on our results, we conducted a sensitivity analysis excluding non-English studies. Data analysis was performed using the Metaprop and Metan Stata packages.

## Results

### Search results

**Fig. 1:**
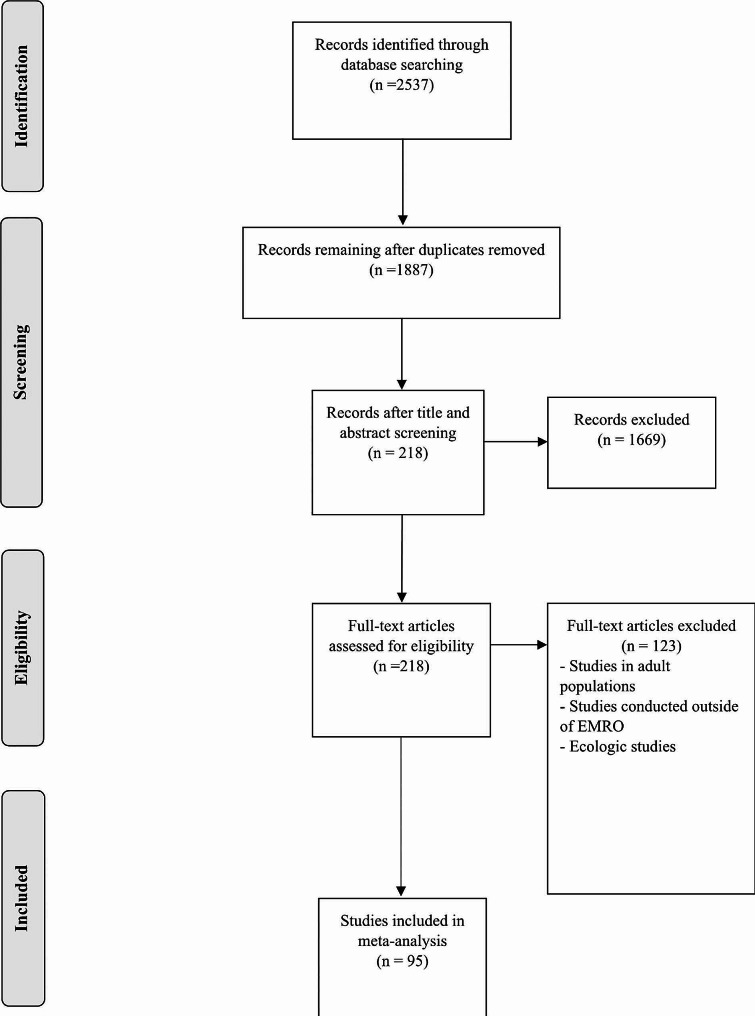
PRISMA flowchart for inclusion process of studies reporting the prevalence of childhood asthma in EMRO

In the first stage, 2537 articles were found; 650 duplicate studies were removed and 1887 studies entered the title and abstract screening phase. Finally, 218 studies were selected to read the full text, 95 of which were eligible to be included in the study, and other studies were excluded for reasons such as inappropriate age range, conducting the study in countries located outside the EMRO region, inappropriate study design and lack of reporting required statistics (Fig. 1).

### Characteristics of included studies

The publication year of the included studies was from 1990 to 2024. A total of 20 countries were included in this meta-analysis. The number of studies included from each country varies, with Iran having the highest number of studies [19], followed by Saudi Arabia [16], Egypt [12], United Arab Emirates [6], Jordan [6] and Pakistan [5]. Some countries have only one study included in the analysis, such as Bahrain and Afghanistan. No study was found from Djibouti and Somalia. One study reported the prevalence of Asthma in both Tunisia and Morocco [20]. Also, one primary international study reporting childhood asthma in the entire EMRO was included in this meta-analysis [13]. Most of the included studies used questionnaires to assess the self-report prevalence of asthma While only 6 studies evaluated the presence of asthma in participants through clinical examinations. The total number of responding participants in the included studies varied from 309 to more than 92,000 individuals. Among the included studies, the study that reported the lowest prevalence of asthma (1%) among children and adolescents was the study of Ahmadiafshar et al., which was conducted in Iran (Zanjan City). The highest asthma prevalence was reported by Alatawi et al., who reported an asthma prevalence of 31.8% in Saudi Arabian participants aged 5–19 years (Appendix, Table B1).

### Risk of bias assessment

The mean JBI score of all included studies was 0.93 ± 0.10 with a minimum of 0.55 and a maximum score of 1 )Appendix, Table B2). The risk of bias was low in most of the included studies, so 86 studies (90.53%) had a low risk of bias, and the risk of bias was high in the remaining 9 studies (9.47%).

### The overall prevalence of asthma in EMRO countries

**Fig. 2:**
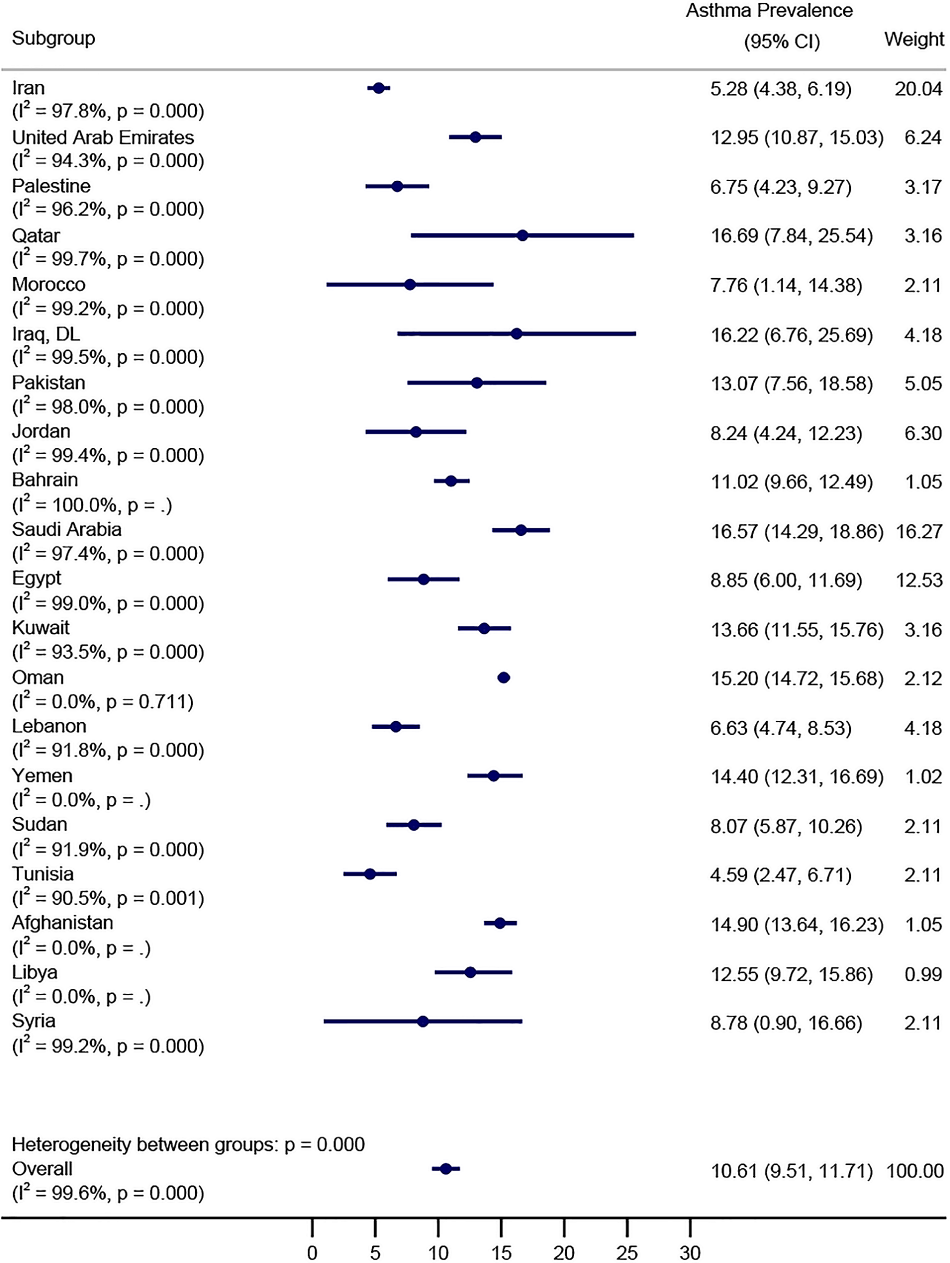
Forest plot illustrating the prevalence of asthma among children and adolescents in the Eastern Mediterranean Region

**Fig. 3:**
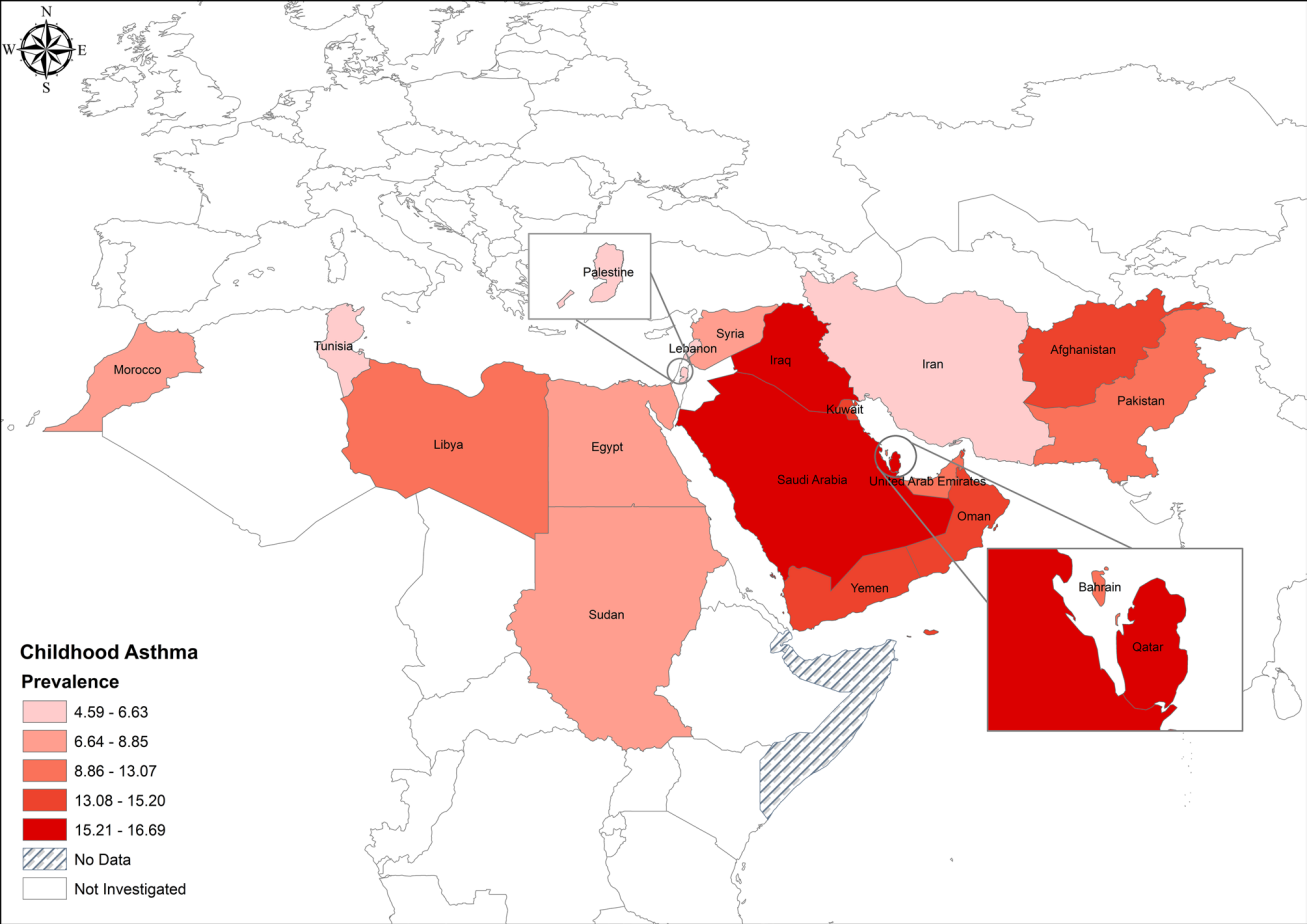
Geographical variation of asthma prevalence among children and adolescents in the Eastern Mediterranean Region

The overall prevalence of asthma in the EMRO countries among a total of 514,468 children and adolescents included in this meta-analysis was 10.61% (95% CI: 9.51–11.71; I^2^: 99.6%; Fig. 2). The country with the highest asthma prevalence was Qatar at 16.69% (95% CI: 7.84–25.54%; I2 = 99.70%), followed closely by Saudi Arabia at 16.57% (95% CI: 14.29–18.86; I2 = 97.40%), and then United Arab Emirates at 12.95% (95% CI: 10.87–15.03; I2 = 94.30%). Iraq (16.22%), Oman (15.20%), and Yemen (14.40%) all fell within the range of 11–16% prevalence rates (Fig. 3). Similarly, Pakistan (13.07%), Kuwait (13.66%), and Libya (12.55%) also had prevalence rates within this range. Bahrain (11.02%), Egypt (8.85%), Jordan (8.24%), and Syria (8.78%) had prevalences ranging from 8 to 11%. Sudan (8.07%) and Morocco (7.76%) had prevalence rates at the lower end of this range. Lebanon (6.64%), Palestine (6.75%), and Tunisia (4.59%) had the lowest prevalence rates, ranging from 4.59 to 6.75%. Iran had one of the lowest prevalence rates at 5.28% (95% CI: 4.38–6.19; I2 = 97.80%).

**Table 1:** Results of subgroup meta-analyses of childhood asthma in EMRO

Variable	Subgroups	Pooled prevalence	95 Confidence interval	I2	Number of studies (References)
Country	Iran	5.28	4.38–6.19	97.80%	19 [21–39]
	United Arab Emirates	12.95	10.87–15.03	94.30%	6 [40–45]
	Palestine	6.75	4.23–9.28	96.20%	3 [46–48]
	Qatar	16.69	7.84–25.54	99.70%	3 [49–51]
	Morocco	7.76	1.14–14.38	99.20%	2 [20, 52]
	Iraq	16.22	6.76–25.69	99.50%	4 [53–56]
	Pakistan	13.07	7.56–18.58	98.00%	5 [57–61]
	Jordan	8.24	4.25–12.23	99.40%	6 [62–67]
	Bahrain	11.02	9.66–12.49	.	1 [68]
	Saudi Arabia	16.57	14.29–18.86	97.40%	16 [16, 69–83]
	Egypt	8.85	6-11.69	99.00%	12 [14, 84–94]
	Kuwait	13.66	11.55–15.76	93.50%	3 [95–97]
	Oman	15.20	14.72–15.68	0.00%	2 [98, 99]
	Lebanon	6.64	4.74–8.53	91.80%	4 [100–103]
	Yemen	14.40	12.31–16.69	.	1 [104]
	Sudan	8.07	5.87–10.26	91.90%	2 [105, 106]
	Tunisia	4.59	2.47–6.71	90.50%	2 [20, 107]
	Afghanistan	14.90	13.64–16.23	.	1 [108]
	Libya	12.55	9.72–15.86	.	1 [109]
	Syria	8.78	0.9-16.66	99.20%	2 [110, 111]
Age group	Children	9.70	8.29–11.10	99.07%	36
	Adolescents	10.10	8.82–11.38	98.93%	48
Gender	Girls	9.75	8.51–10.99	99.07%	56
	Boys	11.48	10.18–12.78	98.47%	57
Place of residence	Rural Area	8.29	6.91–9.68	96.62%	13
	Urban Area	11.27	9.32–13.22	98.76%	22
Publication year	1990s	10.359	5.87–14.85	99.50%	4
	2000s	10.323	8.59–12.05	99.20%	30
	2010s	10.487	8.62–12.36	99.70%	40
	2020s	11.646	9.39–13.9	99.10%	21
Sample size	0-4999	10.99	9.84–12.14	98.80%	76
	5000–10,000	9.22	6.13–12.31	99.70%	11
	> 10,000	9.549	5.29–13.8	99.90%	8
Assessment method	Clinical examination	15.813	7.78–23.85	100.00%	6
	Self-report	10.273	9.37–11.18	99.20%	89
Risk of bias	High risk	9.986	6.86–13.11	99.00%	9
	Low risk	10.751	9.57–11.94	99.60%	86

### Overall prevalence of asthma according to gender, age and place of residence

The prevalence of asthma in girls was lower than that of boys under study, with the pooled prevalence of asthma among girls in EMRO countries estimated at 9.75% (95% CI: 8.51–10.99; I2: 99.07%), compared to 11.48% (95% CI: 10.18–12.78; I2: 98.47%) in boys (Appendix, Figure C1 & C2). Additionally, the prevalence of asthma differed among different age groups. The pooled prevalence of asthma in children under 10 years old was 9.70% (95% CI: 8.29–11.10; I2: 99.07%), whereas in adolescents aged between 10 and 19 years old, it was slightly higher at 10.10% (95% CI: 8.82–11.38; I2: 98.93%; Figure C3 & C4). Furthermore, the prevalence of asthma varied based on the place of residence. In 13 studies reporting the prevalence of asthma in rural areas, the overall prevalence was 8.29% (95% CI: 6.91–9.68; I2: 96.62%). Conversely, the overall prevalence of asthma in urban areas was estimated to be higher, with a pooled prevalence of 11.27% (95% CI: 9.32–13.22; I2: 98.76%; Figure C5 & C6).

### Subgroup analysis based on assessment method, risk of bias, publication year, and sample size

In studies that used clinical examinations to evaluate the prevalence of asthma, the pooled prevalence was found to be 15.81% (95% CI: 7.78–23.85; I2: 100.00%). Conversely, self-reporting resulted in a higher pooled prevalence of asthma, at 10.27% (95% CI: 9.37–11.18; I2: 99.20%). Asthma prevalence among studies with a high risk of bias was 9.986% (95% CI: 6.86–13.11; I2: 99.00%), while among studies with a low risk of bias, it was slightly higher at 10.751% (95% CI: 9.57–11.94; I2: 99.60%).

In terms of publication year, the pooled prevalence of asthma in the 1990s was 10.359% (95% CI: 5.87–14.85; I2: 99.50%) across 4 studies. This prevalence decreased in the 2000s to 10.323% (95% CI: 8.59–12.05; I2: 99.20%) across 30 studies. However, in the 2010s, the prevalence slightly increased to 10.487% (95% CI: 8.62–12.36; I2: 99.70%) across 40 studies. Finally, in the 2020s, the prevalence further increased to 11.646% (95% CI: 9.39–13.9; I2: 99.10%) across 21 studies (Table 1).

Considering sample size, the pooled prevalence of asthma in studies with a sample size greater than 10,000 was 9.549% (95% CI: 5.29–13.8; I2: 99.90%) across 8 studies. For studies with a sample size between 5,000 and 10,000, the pooled prevalence was 9.22% (95% CI: 6.13–12.31; I2: 99.70%) across 11 studies. Finally, studies with a sample size of less than 5,000 showed a pooled prevalence of 10.99% (95% CI: 9.84–12.14; I2: 98.80%) across 76 studies.

### Meta-regression results

A total of 94 studies were included in DerSimonian-Laird random effect meta-regression (Table 2). The overall model fit was moderate with an R-squared value of 53.42% indicating that 53.42% of heterogeneity is explained by the variables included in the model. The Wald chi-square test showed that the model as a whole was statistically significant (*p* < 0.001). In the meta-regression analysis, several variables were examined to assess their impact on the prevalence of asthma across different settings and time periods. Notably, the prevalence of asthma varied significantly across decades, with a substantial increase observed in the 2010s (coefficient: 3.06, p-value: 0.19) and 2020s (coefficient: 3.34, p-value: 0.16) compared to the reference period in the 1990s. Moreover, certain countries exhibited notably higher prevalence rates, particularly Qatar (coefficient: 11.56, p-value: 0.00), Iraq (coefficient: 11.05, p-value: 0.00), and Saudi Arabia (coefficient: 11.14, p-value: 0.00). Conversely, lower prevalence rates were observed in countries such as Iran, where the coefficient for asthma prevalence was not statistically significant compared to the reference. Additionally, the analysis considered the influence of sample size and risk of bias, although the associations were not statistically significant for these factors.

**Table 2:** Meta-regression results for prevalence of childhood asthma in EMRO

Variable		Coefficient	Standard error	*P*-value	Lower limit	Upper limit
Risk of bias	High risk	Ref	-	-	-	-
	Low risk	1.37	1.58	0.39	-1.73	4.48
Decade	1990s	Ref	-	-	-	-
	2000s	1.33	2.35	0.57	-3.27	5.93
	2010s	3.06	2.34	0.19	-1.54	7.65
	2020s	3.34	2.39	0.16	-1.33	8.02
Sample size	< 5000	Ref				
	5000–10,000	-1.41	1.49	0.35	-4.33	1.52
	> 10,000	-1.71	1.89	0.37	-5.40	1.99
Country	Iran	Ref	-	-	-	-
	United Arab Emirates	8.21	1.96	0.00	4.37	12.05
	Palestine	2.84	2.71	0.29	-2.46	8.14
	Qatar	11.56	2.57	0.00	6.52	16.60
	Morocco	8.08	4.31	0.06	-0.36	16.52
	Iraq	11.05	2.28	0.00	6.59	15.52
	Pakistan	7.68	2.11	0.00	3.55	11.82
	Jordan	3.43	1.92	0.08	-0.34	7.20
	Bahrain	4.83	4.14	0.24	-3.28	12.94
	Saudi Arabia	11.14	1.40	0.00	8.40	13.87
	Egypt	2.85	1.52	0.06	-0.12	5.82
	Kuwait	10.74	2.76	0.00	5.33	16.16
	Oman	12.26	3.25	0.00	5.89	18.62
	Lebanon	2.10	2.35	0.37	-2.50	6.70
	Yemen	8.21	4.22	0.05	-0.07	16.48
	Sudan	2.74	2.98	0.36	-3.10	8.59
	Tunisia	0.17	3.07	0.96	-5.85	6.20
	Afghanistan	8.71	4.13	0.04	0.62	16.80
	Libya	6.07	4.43	0.17	-2.61	14.76
	Syria	3.28	3.12	0.29	-2.82	9.39
Constant		1.76	2.84	0.54	-3.81	7.33

### Publication bias

**Fig. 4:**
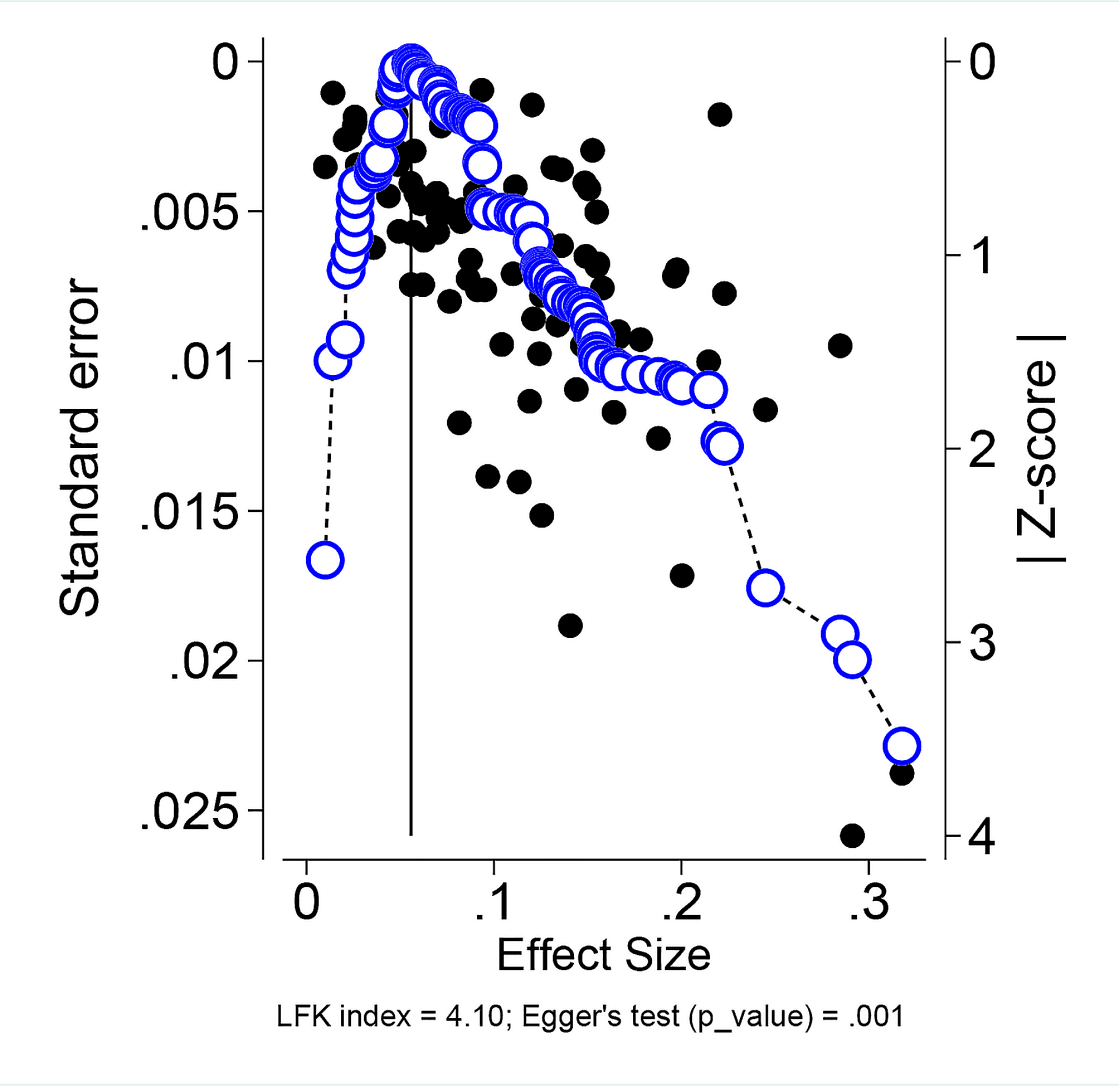
Asymmetric Doi plot revealing the probability of publication bias in asthma prevalence among children and adolescents across EMRO countries

Egger’s test results indicated that publication bias may exist in this study (p-value = 0.01), and these findings were consistent with the Doi plot which had an asymmetric shape (Fig. 4). In the nonparametric trim-and-fill analysis of publication bias, 11 studies on the left side were imputed to address potential bias. The observed prevalence was 10.6%, while the adjusted prevalence, including imputed studies, was 9.05%. The sensitivity analysis, which excluded non-English (Persian) studies, yielded results consistent with the main analysis (Figure C7).

## Discussion

### Asthma prevalence in EMRO: unraveling disparities

The overall prevalence of asthma in the Eastern Mediterranean Regional Office (EMRO) countries, as determined by our meta-analysis, was found to be 10.61% (95% CI: 9.51–11.71; I2: 99.6%). This estimate was derived from a comprehensive analysis of data from a total of 514,468 children and adolescents included in the study. Interestingly, our findings reveal a slightly higher prevalence compared to the prevalence reported by Mallol et al. in 2013, which was 9.35%, contrasting with the 8% prevalence of asthma reported by the WHO for the EMRO [13, 112]. This discrepancy may stem from variations in study methodologies, population demographics, or changes in asthma prevalence over time. On the other hand, in conjunction with the Doi plot and trim-and-fill analysis, it’s important to acknowledge that our findings may still be influenced by publication bias, wherein studies reporting higher prevalence rates of asthma are more likely to be published, potentially leading to an overestimation of asthma prevalence in the EMRO countries. One possible explanation for the higher prevalence in our study is that childhood asthma prevalence was relatively higher in the 2020s compared to previous decades, aligning with WHO projections of asthma deaths in the Eastern Mediterranean Region, estimated at 20,000 in 2015 and anticipated to reach 27,000 by 2030 [112].

This study highlights a significant disparity in asthma prevalence between Arab and African countries within the Eastern Mediterranean Regional Office (EMRO) region, suggesting potential discrepancies in reporting and diagnosis practices. The prevalence of asthma in Arab countries, including Qatar, Saudi Arabia, and the United Arab Emirates, exhibited notably higher rates ranging from 12.95 to 16.69%, compared to African countries such as Sudan and Morocco, where prevalence rates fell within the lower range of 7.76–8.07%. Some of this discrepancy may be attributed to underreporting and underdiagnosis of asthma in African countries, as indicated in previous studies, which is consistent with the weaker body of evidence identified in African countries revealed by the current meta-analysis [113, 114].

Urbanization is another factor that might cause a higher prevalence in Arab countries compared to African ones. In our study, we found a significantly higher prevalence of asthma in urban areas compared to rural ones, with rates of 11.27% and 8.29%, respectively. This difference can be attributed to several factors inherent to urban environments, including heightened levels of both indoor and outdoor air pollution, which introduce harmful substances like particulate matter, nitrogen dioxide (NO2), and ozone. Additionally, urban dwellings often face issues related to pest infestations and mold growth, particularly in substandard housing conditions, serving as potent triggers for asthma exacerbations. Moreover, the presence of endotoxins in urban environments, particularly in poorly maintained housing, further compounds the problem. Economic disparities and housing inadequacies prevalent in urban neighborhoods can exacerbate exposure to asthma triggers like pests, mold, and indoor pollutants. Furthermore, the prevalence of obesity and chronic stress, more common among urban children living in poverty, serves to worsen asthma outcomes, underscoring the multifaceted nature of the urban asthma burden [115–118].

Environmental factors beyond air pollution, like pollen, dust mites, and pet dander, play a significant role in triggering asthma symptoms, contributing to the variation in asthma prevalence observed across different countries [119, 120]. Exposure to dust particles has been implicated in respiratory diseases, including asthma and pneumonia [121]. This is exacerbated by the emergence of new dust source regions in countries such as Iraq, Syria, Jordan, and Saudi Arabia over the last few decades. These regions, characterized by dry river beds (wadis) and lakes, contribute to the proliferation of easily erodible materials, intensifying the occurrence of dust storms [122]. Therefore, these dust storms could also contribute to the disparity in childhood asthma prevalence observed in the region.

Cultural and socioeconomic differences among EMRO countries are a key factor contributing to the diversity observed in studies within the region. One such cultural aspect is the variation in breastfeeding practices. For instance, African nations like Ethiopia (58.2%) and Tanzania (52.6%) demonstrate higher rates of breastfeeding, while countries such as Saudi Arabia (27.6%) and Qatar (24.3%) report notably lower prevalence rates [123–125]. A meta-analysis has illuminated the protective impact of breastfeeding against childhood asthma, indicating a potential risk reduction of up to 16%. Thus, it is plausible that a portion of the higher prevalence rates in Arab countries could be attributed to this factor [126]. Exposure to active smoking and passive smoking is strongly associated with pediatric asthma. Children who reported smoking 300 or more cigarettes per year had a relative risk of 3.9 for new-onset asthma compared with nonsmokers. Moreover, children living with either outdoor smokers, where the odds of asthma are 1.27 times higher, or indoor smokers, where the odds rise to 1.46, exhibit elevated risk. Additionally, regular smoking is linked to an increased likelihood of new-onset asthma [127, 128]. The overall median prevalence of secondhand smoking exposure at home reported across eight Sub-Saharan African countries is 13.8% [129]. Notably, in countries like Qatar, Saudi Arabia, Pakistan, and Kuwait, this prevalence was even higher, reaching 19.3%, 26.4%, 34.3%, and 45.8–51.6% respectively [130–133]. This increased exposure to secondhand smoke may contribute to the elevated prevalence of asthma in these regions, impacting not only children but also pregnant women. Studies indicate that maternal secondhand smoke exposure during pregnancy is associated with an enhanced risk of childhood asthma development [134].

The heterogeneity of asthma prevalence within populations, particularly in regions like the EMRO, may be influenced by a complex interplay of genetic and environmental factors. Genome-Wide Association Studies (GWAS) have unveiled a multitude of genes linked to asthma, shedding light on potential genetic predispositions. For instance, studies such as the GABRIEL study (a multidisciplinary study to identify the genetic and environmental causes of asthma in the European Community) have pinpointed genes on various chromosomes, such as 2, 6, 9, 15, 17, and 22, associated with asthma development. Notably, the Sphingolipid Biosynthesis Regulator 3 (ORMDL3) gene on chromosome 17 has been implicated in childhood-onset asthma, while the Human Leukocyte Antigen DQ (HLA-DQ) gene has been associated with later-onset asthma [135]. These findings underscore the genetic component of asthma susceptibility. However, the manifestation of asthma is not solely dictated by genetic makeup; environmental influences also play a crucial role. Factors like air pollution and tobacco smoke exposure can exacerbate asthma symptoms, particularly in individuals with genetic susceptibilities [136]. This interaction between genetic predispositions and environmental exposures adds complexity to the understanding of asthma heterogeneity, emphasizing the need for comprehensive approaches that consider both genetic and environmental factors in asthma research and healthcare interventions within the EMRO region and beyond.

### Insights for future research

While our efforts aimed to elucidate the variance in asthma prevalence across EMRO countries and the associated risk factors, quantifying the specific contribution of these factors to asthma prevalence in each country remains challenging. Tailored strategies should be devised for individual countries to alleviate asthma prevalence effectively. Future research should concentrate on estimating epidemiological measures such as population-attributable fractions to discern the extent to which each factor contributes to asthma prevalence. Additionally, conducting cost-effectiveness analyses will aid in prioritizing strategies aimed at reducing asthma prevalence efficiently.

### Limitations

It is essential to acknowledge the limitations of our study when interpreting the results. The included studies exhibited significant heterogeneity, which may affect the overall estimates. Additionally, relying on self-report questionnaires and clinical examinations to assess asthma prevalence may introduce measurement bias and misclassification, potentially affecting the accuracy of our estimates. There is also the possibility of publication bias, where studies with significant findings are more likely to be published, thus potentially skewing the overall prevalence estimates. Although there were no language restrictions, all included studies were either in Persian or English. To assess the potential impact of this language distribution, a sensitivity analysis was conducted to examine whether the increased weight of studies from Iran affected the results. The sensitivity analysis yielded similar findings to the main analysis, underscoring the robustness of our conclusions in this regard. However, the EMRO region consists of countries with diverse socioeconomic, environmental, and healthcare contexts, which may influence prevalence estimates and limit the generalizability of our findings. Furthermore, data availability from some countries may be limited, impacting the representativeness of our analysis for certain regions. Finally, while we examined prevalence trends over different decades, we did not fully explore the potential impact of changes in diagnostic criteria, awareness, and reporting practices over time. Nevertheless, our systematic review and meta-analysis offer valuable insights into the prevalence of asthma among children and adolescents in the EMRO region. These findings contribute to the existing knowledge on this topic and highlight the necessity for further research to explore the complex factors that influence asthma prevalence in this specific area.

## Conclusion

Efforts to reduce asthma prevalence in Arab countries and address underdiagnosis in African nations within the EMRO are imperative. In Arab countries with higher asthma rates, interventions must target environmental triggers like air pollution and indoor allergens, alongside improving access to healthcare and raising awareness about asthma management. Concurrently, African countries require improved diagnostic capabilities and healthcare infrastructure to ensure accurate identification and treatment of asthma cases. This necessitates collaborative action among governments, healthcare providers, researchers, and community organizations. By implementing targeted measures, we can alleviate the burden of asthma, enhance respiratory health, and promote equitable access to asthma care across the EMRO region.

### Electronic supplementary material

Below is the link to the electronic supplementary material.

Supplementary Material 1

## Data Availability

Data will be made available on request.

## Abbreviations

EMRO: Eastern Mediterranean Regional Office
ISAAC: International study of asthma and allergies in childhood
WHO: World health organization
GWAS: Genome-wide association studies
NO2: Nitrogen dioxide
JBI: Joanna Briggs Institute
PRISMA: Preferred reporting items for systematic reviews and meta-analyses
PROSPERO: International prospective register of systematic reviews
CI: Confidence interval
ORMDL3: Sphingolipid biosynthesis regulator 3
HLA-DQ: Human leukocyte antigen DQ
