# Supplementary material for: Prevalence of asthma among children and adolescents in WHO’s Eastern Mediterranean Region: a meta-analysis of over 0.5 million participants
Source: BMC Public Health. 2024 Aug 7;24:2148. doi: 10.1186/s12889-024-18716-2 (PMC11308745; doi:10.1186/s12889-024-18716-2)
Supplement: Supplementary file 1 — Supplementary Material 1 [file 12889_2024_18716_MOESM1_ESM.docx]

**Appendix A: Search Strategy for Databases**

**Pubmed:**

1. Prevalence[Mesh] OR Prevalence[TIAB] OR Epidemiology[Mesh] OR Epidemiology[TIAB]

2. Asthma[Mesh] OR Asthma∗[TIAB]

3. Child[Mesh] OR Child∗[TIAB] OR “Adult Children”[Mesh] OR “Adult Children”[TIAB] OR Pediatrics[Mesh] OR Pediatric∗[TIAB] OR Adolescent*[TIAB] OR Teen*[tiab] OR Teenager*[tiab] OR Youth*[tiab] OR juvenile[tiab]

4. Afghanistan[Mesh] OR Bahrain[Mesh] OR Djibouti[Mesh] OR Egypt[Mesh] OR Iran[Mesh] OR Iraq[Mesh] OR Jordan[Mesh] OR Kuwait[Mesh] OR Lebanon[Mesh] OR Libya[Mesh] OR Morocco[Mesh] OR Oman[Mesh] OR Pakistan[Mesh] OR Qatar[Mesh] OR Somalia[Mesh] OR Sudan[Mesh] OR Syria[Mesh] OR Tunisia[Mesh] OR "United Arab Emirates"[Mesh] OR Yemen[Mesh] OR Afghanistan[tw] OR Bahrain[tw] OR Djibouti[tw] OR Egypt[tw] OR Iran[tw] OR Iraq[tw] OR Jordan[tw] OR Kuwait[tw] OR Lebanon[tw] OR Libya[tw] OR Morocco[tw] OR Oman[tw] OR Pakistan[tw] OR Qatar[tw] OR Somalia[tw] OR Sudan[tw] OR Syria*[tw] OR Tunisia[tw] OR "United Arab Emirates"[tw] OR "UAE"[tw] OR Yemen[tw] OR "Eastern Mediterranean"[tw]

5. Kabul[tw] OR Kandahar[tw] OR Herat[tw] OR Mazar-i-Sharif[tw] OR Kunduz[tw] OR Jalalabad[tw] OR Taloqan[tw] OR Puli Khumri[tw] OR Charikar[tw] OR Lashkargah[tw] OR Sheberghan[tw] OR Ghazni[tw] OR Khost[tw] OR Sar-e Pol[tw] OR Chaghcharan[tw] OR Mihtarlam[tw] OR Farah[tw] OR Puli Alam[tw] OR Manama[tw] OR Riffa[tw] OR Muharraq[tw] OR Hamad Town[tw] OR A'ali[tw] OR Isa Town[tw] OR Sitra[tw] OR Budaiya[tw] OR Jidhafs[tw] OR Al-Malikiyah[tw] OR Cairo[tw] OR Alexandria[tw] OR Giza[tw] OR Shubra El Kheima[tw] OR “Port Said”[tw] OR Suez[tw] OR “El Mahalla”[tw] OR “El Kubra”[tw] OR Luxor[tw] OR Mansoura[tw] OR Tanta[tw] OR Asyut[tw] OR Ismailia[tw] OR Faiyum[tw] OR Zagazig[tw] OR Damietta[tw] OR Minya[tw] OR Damanhur[tw] OR “Beni Suef”[tw] OR Hurghada[tw] OR Qena[tw] OR Sohag[tw] OR “Shibin El Kom”[tw] OR Banha[tw] OR Arish[tw] OR Mashhad[tw] OR Esfahan[tw] OR Shiraz[tw] OR Tabriz[tw] OR[tw] OR Karaj[tw] OR Qom[tw] OR Ahvaz[tw] OR Kermanshah[tw] OR[tw] ORumiyeh[tw] OR Rasht[tw] OR Bandar-Abbas[tw] OR Zahedan[tw] OR Hamadan[tw] OR Ardabil[tw] OR Yazd[tw] OR Arak[tw] OR Kerman[tw] OR Eslamshahr[tw] OR Zanjan[tw] OR Sanandaj[tw] OR Qazvin[tw] OR Khorramabad[tw] OR Shahriyar[tw] OR Gorgan[tw] OR Qods[tw] OR Kashan[tw] OR Sari[tw] OR Baghdad[tw] OR Mosul[tw] OR Basra[tw] OR Nasiriyah[tw] OR Hillah[tw] OR Suleymaniyah[tw] OR Erbil[tw] OR Ramadi[tw] OR Baqubah[tw] OR Kirkuk[tw] OR Tikrit[tw] OR Najaf[tw] OR Kut[tw] OR Dohuk[tw] OR Diwaniyah[tw] OR Karbala[tw] OR Zakho[tw] OR Amarah[tw] OR Samawah[tw] OR Samarra[tw] OR Fallujah[tw] OR Halabja[tw] OR Kufa[tw] OR Khanaqin[tw] OR “Tal Afar”[tw] OR “Tuz Khurmatu”[tw] OR Amman[tw] OR Zarqa[tw] OR Irbid[tw] OR Russeifa[tw] OR Sahab[tw] OR Ar Ramtha[tw] OR “Al Fahahil”[tw] OR Kuwait City[tw] OR Ar Riqqah[tw] OR “Al Manqaf”[tw] OR “Al Jahra”[tw] OR “Al Fintas”[tw] OR Janub[tw] OR Surra[tw] OR Beirut[tw] OR Tripoli[tw] OR Sidon[tw] OR Tyre[tw] OR Baalbek[tw] OR Nabatieh[tw] OR Aley[tw] OR Jounieh[tw] OR Zahle[tw] OR Zgharta-Ehden[tw] OR Byblos[tw] OR Batroun[tw] OR Tripoli[tw] OR Benghazi[tw] OR Misrata[tw] OR “Al Bayda”[tw] OR Zawiya[tw] OR Zliten[tw] OR Tobruk[tw] OR Ajdabiya[tw] OR Derna[tw] OR Sabha[tw] OR Khoms[tw] OR Fez[tw] OR Tangier[tw] OR Marrakesh[tw] OR Salé[tw] OR Meknes[tw] OR Rabat[tw] OR Oujda[tw] OR Kenitra[tw] OR Agadir[tw] OR Tetouan[tw] OR Temara[tw] OR Safi[tw] OR Mohammedia[tw] OR Khouribga[tw] OR “El Jadida”[tw] OR “Beni Mellal”[tw] OR “Aït Melloul”[tw] OR Nador[tw] OR “Dar Bouazza”[tw] OR Taza[tw] OR Settat[tw] OR Berrechid[tw] OR Khemisset[tw] OR Inezgane[tw] OR “Ksar El Kebir”[tw] OR Larache[tw] OR Guelmim[tw] OR Khenifra[tw] OR Berkane[tw] OR Taourirt[tw] OR Bouskoura[tw] OR Karachi[tw] OR Lahore[tw] OR Faisalabad[tw] OR Rawalpindi[tw] OR Gujranwala[tw] OR Peshawar[tw] OR Multan[tw] OR Hyderabad[tw] OR Islamabad[tw] OR Quetta[tw] OR Bahawalpur[tw] OR Sargodha[tw] OR Sialkot[tw] OR Sukkur[tw] OR Larkana[tw] OR “Rahim Yar Khan”[tw] OR Sheikhupura[tw] OR Jhang[tw] OR Talagang[tw] OR “Dera Ghazi Khan”[tw] OR Gujrat[tw] OR Sahiwal[tw] OR “Wah Cantonment”[tw] OR Mardan[tw] OR Kasur[tw] OR Okara[tw] OR Mingora[tw] OR Nawabshah[tw] OR Chiniot[tw] OR Kotri[tw] OR Kāmoke[tw] OR Hafizabad[tw] OR Sadiqabad[tw] OR Mirpur Khas[tw] OR Burewala[tw] OR Kohat[tw] OR Khanewal[tw] OR” Dera Ismail Khan”[tw] OR Turbat[tw] OR Muzaffargarh[tw] OR Abbotabad[tw] OR “Mandi Bahauddin”[tw] OR Shikarpur[tw] OR Jacobabad[tw] OR Jhelum[tw] OR Khanpur[tw] OR Khairpur[tw] OR Khuzdar[tw] OR Pakpattan[tw] OR Daska[tw] OR Gojra[tw] OR Dadu[tw] OR Muridke[tw] OR Bahawalnagar[tw] OR Samundri[tw] OR Tando Allahyar[tw] OR Tando Adam[tw] OR Jaranwala[tw] OR Chishtian[tw] OR Muzaffarabad[tw] OR Doha[tw] OR “Al Rayyan” Mogadishu[tw] OR Hargeysa[tw] OR Borama[tw] OR Kismayo[tw] OR Merca[tw] OR Jamame[tw] OR Baidoa[tw] OR Burao[tw] OR Bosaso[tw] OR Khartoum[tw] OR Omdurman[tw] OR “Khartoum North”[tw] OR Kassala[tw] OR “Port Sudan”[tw] OR “El Obeid”[tw] OR Gedaref[tw] OR Aleppo[tw] OR Damascus[tw] OR Daraa[tw] OR “Deir ez-Zor”[tw] OR Hama[tw] OR Al-Hasakah[tw] OR Homs[tw] OR Idlib[tw] OR Latakia[tw] OR Quneitra[tw] OR Raqqa[tw] OR As-Suwayda[tw] OR Tartus[tw] OR Dubai[tw] OR “Abu Dhabi”[tw] OR Sharjah[tw] OR “Al Ain”[tw] OR Ajman[tw] OR “Ras Al Khaimah”[tw] OR Fujairah[tw] OR Sana'a[tw] OR Ta'izz[tw] OR “Al Hudaydah”[tw] OR Aden[tw] OR Ibb[tw] OR Dhamar[tw] OR Mukalla[tw] OR Seiyun[tw]

6. #4 OR #5

7. #1 AND #2 AND #3 AND #6

**Scopus:**

1. TITLE-ABS-KEY(Prevalence OR Epidemiology)
2. TITLE-ABS-KEY(Asthma*)
3. TITLE-ABS-KEY(Child∗ OR “Adult Children” OR “Adult Children” OR Pediatric∗ OR Adolescent* OR Teen* OR Teenager* OR Youth* OR juvenile)
4. TITLE-ABS-KEY(Afghanistan OR Bahrain OR Djibouti OR Egypt OR Iran OR Iraq OR Jordan OR Kuwait OR Lebanon OR Libya OR Morocco OR Oman OR Pakistan OR Qatar OR Somalia OR Sudan OR Syria OR Tunisia OR "United Arab Emirates" OR Yemen "Eastern Mediterranean")
5. AFFILCOUNTRY ( Afghanistan OR Bahrain OR Djibouti OR Egypt OR Iran OR Iraq OR Jordan OR Kuwait OR Lebanon OR libya OR Morocco OR Oman OR pakistan OR Qatar OR Somalia OR Sudan OR Syria OR Tunisia OR "United Arab Emirates" OR Yemen)
6. TITLE-ABS-KEY (Kabul OR Kandahar OR Herat OR Mazar-i-Sharif OR Kunduz OR Jalalabad OR Taloqan OR Puli Khumri OR Charikar OR Lashkargah OR Sheberghan OR Ghazni OR Khost OR Sar-e Pol OR Chaghcharan OR Mihtarlam OR Farah OR Puli Alam OR Manama OR Riffa OR Muharraq OR Hamad Town OR A'ali OR Isa Town OR Sitra OR Budaiya OR Jidhafs OR Al-Malikiyah OR Cairo OR Alexandria OR Giza OR Shubra El Kheima OR “Port Said” OR Suez OR “El Mahalla” OR “El Kubra” OR Luxor OR Mansoura OR Tanta OR Asyut OR Ismailia OR Faiyum OR Zagazig OR Damietta OR Minya OR Damanhur OR “Beni Suef” OR Hurghada OR Qena OR Sohag OR “Shibin El Kom” OR Banha OR Arish OR Mashhad OR Esfahan OR Shiraz OR Tabriz OR Karaj OR Qom OR Ahvaz OR Kermanshah OR Orumiyeh OR Rasht OR Bandar-Abbas OR Zahedan OR Hamadan OR Ardabil OR Yazd OR Arak OR Kerman OR Eslamshahr OR Zanjan OR Sanandaj OR Qazvin OR Khorramabad OR Shahriyar OR Gorgan OR Qods OR Kashan OR Sari OR Baghdad OR Mosul OR Basra OR Nasiriyah OR Hillah OR Suleymaniyah OR Erbil OR Ramadi OR Baqubah OR Kirkuk OR Tikrit OR Najaf OR Kut OR Dohuk OR Diwaniyah OR Karbala OR Zakho OR Amarah OR Samawah OR Samarra OR Fallujah OR Halabja OR Kufa OR Khanaqin OR “Tal Afar” OR “Tuz Khurmatu” OR Amman OR Zarqa OR Irbid OR Russeifa OR Sahab OR Ar Ramtha OR “Al Fahahil” OR Kuwait City OR Ar Riqqah OR “Al Manqaf” OR “Al Jahra” OR “Al Fintas” OR Janub OR Surra OR Beirut OR Tripoli OR Sidon OR Tyre OR Baalbek OR Nabatieh OR Aley OR Jounieh OR Zahle OR Zgharta-Ehden OR Byblos OR Batroun OR Tripoli OR Benghazi OR Misrata OR “Al Bayda” OR Zawiya OR Zliten OR Tobruk OR Ajdabiya OR Derna OR Sabha OR Khoms OR Fez OR Tangier OR Marrakesh OR Salé OR Meknes OR Rabat OR Oujda OR Kenitra OR Agadir OR Tetouan OR Temara OR Safi OR Mohammedia OR Khouribga OR “El Jadida” OR “Beni Mellal” OR “Aït Melloul” OR Nador OR “Dar Bouazza” OR Taza OR Settat OR Berrechid OR Khemisset OR Inezgane OR “Ksar El Kebir” OR Larache OR Guelmim OR Khenifra OR Berkane OR Taourirt OR Bouskoura OR Karachi OR Lahore OR Faisalabad OR Rawalpindi OR Gujranwala OR Peshawar OR Multan OR Hyderabad OR Islamabad OR Quetta OR Bahawalpur OR Sargodha OR Sialkot OR Sukkur OR Larkana OR “Rahim Yar Khan” OR Sheikhupura OR Jhang OR Talagang OR “Dera Ghazi Khan” OR Gujrat OR Sahiwal OR “Wah Cantonment” OR Mardan OR Kasur OR Okara OR Mingora OR Nawabshah OR Chiniot OR Kotri OR Kāmoke OR Hafizabad OR Sadiqabad OR Mirpur Khas OR Burewala OR Kohat OR Khanewal OR” Dera Ismail Khan” OR Turbat OR Muzaffargarh OR Abbotabad OR “Mandi Bahauddin” OR Shikarpur OR Jacobabad OR Jhelum OR Khanpur OR Khairpur OR Khuzdar OR Pakpattan OR Daska OR Gojra OR Dadu OR Muridke OR Bahawalnagar OR Samundri OR Tando Allahyar OR Tando Adam OR Jaranwala OR Chishtian OR Muzaffarabad OR Doha OR “Al Rayyan” Mogadishu OR Hargeysa OR Borama OR Kismayo OR Merca OR Jamame OR Baidoa OR Burao OR Bosaso OR Khartoum OR Omdurman OR “Khartoum North” OR Kassala OR “Port Sudan” OR “El Obeid” OR Gedaref OR Aleppo OR Damascus OR Daraa OR “Deir ez-Zor” OR Hama OR Al-Hasakah OR Homs OR Idlib OR Latakia OR Quneitra OR Raqqa OR As-Suwayda OR Tartus OR Dubai OR “Abu Dhabi” OR Sharjah OR “Al Ain” OR Ajman OR “Ras Al Khaimah” OR Fujairah OR Sana'a OR Ta'izz OR “Al Hudaydah” OR Aden OR Ibb OR Dhamar OR Mukalla OR Seiyun)
7. #4 OR #5 OR #6
8. #1 AND #2 AND #3 AND #7

**Embase:**

1. “Prevalence”/exp OR Prevalence:ti,ab OR “Epidemiology”/exp OR Epidemiology:ti,ab
2. “Asthma”/exp OR Asthma∗:ti,ab
3. “Child”/exp OR Child∗:ti,ab OR “Adult Children”/exp OR “Adult Children”:ti,ab OR “Pediatrics”/exp OR Pediatric∗:ti,ab OR Adolescent*:ti,ab OR Teen*:ti,ab OR Teenager*:ti,ab OR Youth*:ti,ab OR juvenile:ti,ab
4. “Afghanistan”/exp OR “Bahrain”/exp OR “Djibouti”/exp OR “Egypt”/exp OR “Iran”/exp OR “Iraq”/exp OR “Jordan”/exp OR “Kuwait”/exp OR “Lebanon”/exp OR “Libya”/exp OR “Morocco”/exp OR “Oman”/exp OR “Pakistan”/exp OR “Qatar”/exp OR “Somalia”/exp OR “Sudan”/exp OR “Syria”/exp OR “Tunisia”/exp OR "United Arab Emirates"/exp OR “Yemen”/exp OR Afghanistan:ti,ab OR Bahrain:ti,ab OR Djibouti:ti,ab OR Egypt:ti,ab OR Iran:ti,ab OR Iraq:ti,ab OR Jordan:ti,ab OR Kuwait:ti,ab OR Lebanon:ti,ab OR Libya:ti,ab OR Morocco:ti,ab OR Oman:ti,ab OR Pakistan:ti,ab OR Qatar:ti,ab OR Somalia:ti,ab OR Sudan:ti,ab OR Syria*:ti,ab OR Tunisia:ti,ab OR "United Arab Emirates":ti,ab OR "UAE":ti,ab OR Yemen:ti,ab OR "Eastern Mediterranean":ti,ab
5. “Kabul“:ti,ab OR “Kandahar“:ti,ab OR “Herat“:ti,ab OR “Mazar-i-Sharif“:ti,ab OR “Kunduz“:ti,ab OR “Jalalabad“:ti,ab OR “Taloqan“:ti,ab OR “Puli Khumri“:ti,ab OR “Charikar“:ti,ab OR “Lashkargah“:ti,ab OR “Sheberghan“:ti,ab OR “Ghazni“:ti,ab OR “Khost“:ti,ab OR “Sar-e Pol“:ti,ab OR “Chaghcharan“:ti,ab OR “Mihtarlam“:ti,ab OR “Farah“:ti,ab OR “Puli Alam“:ti,ab OR “Manama“:ti,ab OR “Riffa“:ti,ab OR “Muharraq“:ti,ab OR “Hamad Town“:ti,ab OR “Aali“:ti,ab OR “Isa Town“:ti,ab OR “Sitra“:ti,ab OR “Budaiya“:ti,ab OR “Jidhafs“:ti,ab OR “Al-Malikiyah“:ti,ab OR “Cairo“:ti,ab OR “Alexandria“:ti,ab OR “Giza“:ti,ab OR “Shubra El Kheima“:ti,ab OR “Port Said“:ti,ab OR “Suez“:ti,ab OR “El Mahalla”:ti,ab OR “El Kubra“:ti,ab OR “Luxor“:ti,ab OR “Mansoura“:ti,ab OR “Tanta“:ti,ab OR “Asyut“:ti,ab OR “Ismailia“:ti,ab OR “Faiyum“:ti,ab OR “Zagazig“:ti,ab OR “Damietta“:ti,ab OR “Minya“:ti,ab OR “Damanhur“:ti,ab OR “Beni Suef“:ti,ab OR “Hurghada“:ti,ab OR “Qena“:ti,ab OR “Sohag“:ti,ab OR “Shibin El Kom“:ti,ab OR “Banha“:ti,ab OR “Arish“:ti,ab OR “Mashhad”:ti,ab OR “Esfahan”:ti,ab OR “Shiraz”:ti,ab OR “Tabriz”:ti,ab OR “OR Karaj”:ti,ab OR “Qom”:ti,ab OR “Ahvaz”:ti,ab OR “Kermanshah”:ti,ab OR “Orumiyeh”:ti,ab OR “Rasht”:ti,ab OR “Bandar-Abbas”:ti,ab OR “Zahedan”:ti,ab OR “Hamadan”:ti,ab OR “Ardabil”:ti,ab OR “Yazd”:ti,ab OR “Arak”:ti,ab OR “Kerman”:ti,ab OR “Eslamshahr”:ti,ab OR “Zanjan”:ti,ab OR “Sanandaj”:ti,ab OR “Qazvin”:ti,ab OR “Khorramabad”:ti,ab OR “Shahriyar”:ti,ab OR “Gorgan”:ti,ab OR “Qods”:ti,ab OR “Kashan”:ti,ab OR “Sari”:ti,ab OR “Baghdad”:ti,ab OR “Mosul”:ti,ab OR “Basra”:ti,ab OR “Nasiriyah”:ti,ab OR “Hillah”:ti,ab OR “Suleymaniyah”:ti,ab OR “Erbil”:ti,ab OR “Ramadi”:ti,ab OR “Baqubah”:ti,ab OR “Kirkuk”:ti,ab OR “Tikrit”:ti,ab OR “Najaf”:ti,ab OR “Kut”:ti,ab OR “Dohuk”:ti,ab OR “Diwaniyah”:ti,ab OR “Karbala”:ti,ab OR “Zakho”:ti,ab OR “Amarah”:ti,ab OR “Samawah”:ti,ab OR “Samarra”:ti,ab OR “Fallujah”:ti,ab OR “Halabja”:ti,ab OR “Kufa”:ti,ab OR “Khanaqin”:ti,ab OR “Tal Afar”:ti,ab OR “Tuz Khurmatu”:ti,ab OR “Amman”:ti,ab OR “Zarqa”:ti,ab OR “Irbid”:ti,ab OR “Russeifa”:ti,ab OR “Sahab”:ti,ab OR “Ar Ramtha”:ti,ab OR “Al Fahahil”:ti,ab OR “Kuwait City”:ti,ab OR “Ar Riqqah”:ti,ab OR “Al Manqaf”:ti,ab OR “Al Jahra”:ti,ab OR “Al Fintas”:ti,ab OR “Janub”:ti,ab OR “Surra”:ti,ab OR “Beirut”:ti,ab OR “Tripoli”:ti,ab OR “Sidon”:ti,ab OR “Tyre”:ti,ab OR “Baalbek”:ti,ab OR “Nabatieh”:ti,ab OR “Aley”:ti,ab OR “Jounieh”:ti,ab OR “Zahle”:ti,ab OR “Zgharta-Ehden”:ti,ab OR “Byblos”:ti,ab OR “Batroun”:ti,ab OR “Tripoli”:ti,ab OR “Benghazi”:ti,ab OR “Misrata”:ti,ab OR “Al Bayda”:ti,ab OR “Zawiya”:ti,ab OR “Zliten”:ti,ab OR “Tobruk”:ti,ab OR “Ajdabiya”:ti,ab OR “Derna”:ti,ab OR “Sabha”:ti,ab OR “Khoms”:ti,ab OR “Fez”:ti,ab OR “Tangier”:ti,ab OR “Marrakesh”:ti,ab OR “Salé”:ti,ab OR “Meknes”:ti,ab OR “Rabat”:ti,ab OR “Oujda”:ti,ab OR “Kenitra”:ti,ab OR “Agadir”:ti,ab OR “Tetouan”:ti,ab OR “Temara”:ti,ab OR “Safi”:ti,ab OR “Mohammedia”:ti,ab OR “Khouribga”:ti,ab OR “El Jadida”:ti,ab OR “Beni Mellal”:ti,ab OR “Aït Melloul”:ti,ab OR “Nador”:ti,ab OR “Dar Bouazza”:ti,ab OR “Taza”:ti,ab OR “Settat”:ti,ab OR “Berrechid”:ti,ab OR “Khemisset”:ti,ab OR “Inezgane”:ti,ab OR “Ksar El Kebir”:ti,ab OR “Larache”:ti,ab OR “Guelmim”:ti,ab OR “Khenifra”:ti,ab OR “Berkane”:ti,ab OR “Taourirt”:ti,ab OR “Bouskoura”:ti,ab OR “Karachi”:ti,ab OR “Lahore”:ti,ab OR “Faisalabad”:ti,ab OR “Rawalpindi”:ti,ab OR “Gujranwala”:ti,ab OR “Peshawar”:ti,ab OR “Multan”:ti,ab OR “Hyderabad”:ti,ab OR “Islamabad”:ti,ab OR “Quetta”:ti,ab OR “Bahawalpur”:ti,ab OR “Sargodha”:ti,ab OR “Sialkot”:ti,ab OR “Sukkur”:ti,ab OR “Larkana”:ti,ab OR “Rahim Yar Khan”:ti,ab OR “Sheikhupura”:ti,ab OR “Jhang”:ti,ab OR “Talagang”:ti,ab OR “Dera Ghazi Khan”:ti,ab OR “Gujrat”:ti,ab OR “Sahiwal”:ti,ab OR “Wah Cantonment”:ti,ab OR “Mardan”:ti,ab OR “Kasur”:ti,ab OR “Okara”:ti,ab OR “Mingora”:ti,ab OR “Nawabshah”:ti,ab OR “Chiniot”:ti,ab OR “Kotri”:ti,ab OR “Kāmoke”:ti,ab OR “Hafizabad”:ti,ab OR “Sadiqabad”:ti,ab OR “Mirpur Khas”:ti,ab OR “Burewala”:ti,ab OR “Kohat”:ti,ab OR “Khanewal”:ti,ab OR “Dera Ismail Khan”:ti,ab OR “Turbat”:ti,ab OR “Muzaffargarh”:ti,ab OR “Abbotabad”:ti,ab OR “Mandi Bahauddin”:ti,ab OR “Shikarpur”:ti,ab OR “Jacobabad”:ti,ab OR “Jhelum”:ti,ab OR “Khanpur”:ti,ab OR “Khairpur”:ti,ab OR “Khuzdar”:ti,ab OR “Pakpattan”:ti,ab OR “Daska”:ti,ab OR “Gojra”:ti,ab OR “Dadu”:ti,ab OR “Muridke”:ti,ab OR “Bahawalnagar”:ti,ab OR “Samundri”:ti,ab OR “Tando Allahyar”:ti,ab OR “Tando Adam”:ti,ab OR “Jaranwala”:ti,ab OR “Chishtian”:ti,ab OR “Muzaffarabad”:ti,ab OR “ Doha”:ti,ab OR “Al Rayyan Mogadishu”:ti,ab OR “Hargeysa”:ti,ab OR “Borama”:ti,ab OR “Kismayo”:ti,ab OR “Merca”:ti,ab OR “Jamame”:ti,ab OR “Baidoa”:ti,ab OR “Burao”:ti,ab OR “Bosaso”:ti,ab OR “Khartoum”:ti,ab OR “Omdurman”:ti,ab OR “Khartoum North”:ti,ab OR “Kassala”:ti,ab OR “Port Sudan”:ti,ab OR “El Obeid”:ti,ab OR “Gedaref”:ti,ab OR “Aleppo”:ti,ab OR “Damascus”:ti,ab OR “Daraa”:ti,ab OR “Deir ez-Zor”:ti,ab OR “Hama”:ti,ab OR “Al-Hasakah”:ti,ab OR “Homs”:ti,ab OR “Idlib”:ti,ab OR “Latakia”:ti,ab OR “Quneitra”:ti,ab OR “Raqqa”:ti,ab OR “As-Suwayda”:ti,ab OR “Tartus”:ti,ab OR “Dubai”:ti,ab OR “Abu Dhabi”:ti,ab OR “Sharjah”:ti,ab OR “Al Ain”:ti,ab OR “Ajman”:ti,ab OR “Ras Al Khaimah”:ti,ab OR “Fujairah”:ti,ab OR “Sanaa”:ti,ab OR “Taizz”:ti,ab OR “Al Hudaydah”:ti,ab OR “Aden”:ti,ab OR “Ibb”:ti,ab OR “Dhamar”:ti,ab OR “Mukalla”:ti,ab OR “Seiyun”:ti,ab

**Web of knowledge:**

1. ALL = (Prevalence OR Epidemiology)
2. ALL = (Asthma*)
3. TS = (Child∗ OR “Adult Children” OR “Adult Children” OR Pediatrics OR Pediatric∗ OR Adolescent* OR Teen* OR Teenager* OR Youth* OR juvenile)
4. ALL = (Afghanistan OR Bahrain OR Djibouti OR Egypt OR Iran OR Iraq OR Jordan OR Kuwait OR Lebanon OR Libya OR Morocco OR Oman OR Pakistan OR Qatar OR Somalia OR Sudan OR Syria OR Tunisia OR "United Arab Emirates" OR Yemen)
5. TS = (“Kabul“ OR “Kandahar“ OR “Herat“ OR “Mazar-i-Sharif“ OR “Kunduz“ OR “Jalalabad“ OR “Taloqan“ OR “Puli Khumri“ OR “Charikar“ OR “Lashkargah“ OR “Sheberghan“ OR “Ghazni“ OR “Khost“ OR “Sar-e Pol“ OR “Chaghcharan“ OR “Mihtarlam“ OR “Farah“ OR “Puli Alam“ OR “Manama“ OR “Riffa“ OR “Muharraq“ OR “Hamad Town“ OR “A'ali“ OR “Isa Town“ OR “Sitra“ OR “Budaiya“ OR “Jidhafs“ OR “Al-Malikiyah“ OR “Cairo“ OR “Alexandria“ OR “Giza“ OR “Shubra El Kheima“ OR “Port Said“ OR “Suez“ OR “El Mahalla” OR “El Kubra“ OR “Luxor“ OR “Mansoura“ OR “Tanta“ OR “Asyut“ OR “Ismailia“ OR “Faiyum“ OR “Zagazig“ OR “Damietta“ OR “Minya“ OR “Damanhur“ OR “Beni Suef“ OR “Hurghada“ OR “Qena“ OR “Sohag“ OR “Shibin El Kom“ OR “Banha“ OR “Arish“ OR “Mashhad” OR “Esfahan” OR “Shiraz” OR “Tabriz” OR “OR Karaj” OR “Qom” OR “Ahvaz” OR “Kermanshah” OR “Orumiyeh” OR “Rasht” OR “Bandar-Abbas” OR “Zahedan” OR “Hamadan” OR “Ardabil” OR “Yazd” OR “Arak” OR “Kerman” OR “Eslamshahr” OR “Zanjan” OR “Sanandaj” OR “Qazvin” OR “Khorramabad” OR “Shahriyar” OR “Gorgan” OR “Qods” OR “Kashan” OR “Sari” OR “Baghdad” OR “Mosul” OR “Basra” OR “Nasiriyah” OR “Hillah” OR “Suleymaniyah” OR “Erbil” OR “Ramadi” OR “Baqubah” OR “Kirkuk” OR “Tikrit” OR “Najaf” OR “Kut” OR “Dohuk” OR “Diwaniyah” OR “Karbala” OR “Zakho” OR “Amarah” OR “Samawah” OR “Samarra” OR “Fallujah” OR “Halabja” OR “Kufa” OR “Khanaqin” OR “Tal Afar” OR “Tuz Khurmatu” OR “Amman” OR “Zarqa” OR “Irbid” OR “Russeifa” OR “Sahab” OR “Ar Ramtha” OR “Al Fahahil” OR “Kuwait City” OR “Ar Riqqah” OR “Al Manqaf” OR “Al Jahra” OR “Al Fintas” OR “Janub” OR “Surra” OR “Beirut” OR “Tripoli” OR “Sidon” OR “Tyre” OR “Baalbek” OR “Nabatieh” OR “Aley” OR “Jounieh” OR “Zahle” OR “Zgharta-Ehden” OR “Byblos” OR “Batroun” OR “Tripoli” OR “Benghazi” OR “Misrata” OR “Al Bayda” OR “Zawiya” OR “Zliten” OR “Tobruk” OR “Ajdabiya” OR “Derna” OR “Sabha” OR “Khoms” OR “Fez” OR “Tangier” OR “Marrakesh” OR “Salé” OR “Meknes” OR “Rabat” OR “Oujda” OR “Kenitra” OR “Agadir” OR “Tetouan” OR “Temara” OR “Safi” OR “Mohammedia” OR “Khouribga” OR “El Jadida” OR “Beni Mellal” OR “Aït Melloul” OR “Nador” OR “Dar Bouazza” OR “Taza” OR “Settat” OR “Berrechid” OR “Khemisset” OR “Inezgane” OR “Ksar El Kebir” OR “Larache” OR “Guelmim” OR “Khenifra” OR “Berkane” OR “Taourirt” OR “Bouskoura” OR “Karachi” OR “Lahore” OR “Faisalabad” OR “Rawalpindi” OR “Gujranwala” OR “Peshawar” OR “Multan” OR “Hyderabad” OR “Islamabad” OR “Quetta” OR “Bahawalpur” OR “Sargodha” OR “Sialkot” OR “Sukkur” OR “Larkana” OR “Rahim Yar Khan” OR “Sheikhupura” OR “Jhang” OR “Talagang” OR “Dera Ghazi Khan” OR “Gujrat” OR “Sahiwal” OR “Wah Cantonment” OR “Mardan” OR “Kasur” OR “Okara” OR “Mingora” OR “Nawabshah” OR “Chiniot” OR “Kotri” OR “Kāmoke” OR “Hafizabad” OR “Sadiqabad” OR “Mirpur Khas” OR “Burewala” OR “Kohat” OR “Khanewal” OR “Dera Ismail Khan” OR “Turbat” OR “Muzaffargarh” OR “Abbotabad” OR “Mandi Bahauddin” OR “Shikarpur” OR “Jacobabad” OR “Jhelum” OR “Khanpur” OR “Khairpur” OR “Khuzdar” OR “Pakpattan” OR “Daska” OR “Gojra” OR “Dadu” OR “Muridke” OR “Bahawalnagar” OR “Samundri” OR “Tando Allahyar” OR “Tando Adam” OR “Jaranwala” OR “Chishtian” OR “Muzaffarabad” OR “ Doha” OR “Al Rayyan Mogadishu” OR “Hargeysa” OR “Borama” OR “Kismayo” OR “Merca” OR “Jamame” OR “Baidoa” OR “Burao” OR “Bosaso” OR “Khartoum” OR “Omdurman” OR “Khartoum North” OR “Kassala” OR “Port Sudan” OR “El Obeid” OR “Gedaref” OR “Aleppo” OR “Damascus” OR “Daraa” OR “Deir ez-Zor” OR “Hama” OR “Al-Hasakah” OR “Homs” OR “Idlib” OR “Latakia” OR “Quneitra” OR “Raqqa” OR “As-Suwayda” OR “Tartus” OR “Dubai” OR “Abu Dhabi” OR “Sharjah” OR “Al Ain” OR “Ajman” OR “Ras Al Khaimah” OR “Fujairah” OR “Sana'a” OR “Ta'izz” OR “Al Hudaydah” OR “Aden” OR “Ibb” OR “Dhamar” OR “Mukalla” OR “Seiyun”)
6. #4 OR #5
7. #1 AND #2 AND #3 AND #6

**Appendix B: Tables**

**Table B1: Characteristics of studies included in the systematic review and meta-analysis of asthma prevalence in WHO Eastern Mediterranean Region countries.**

| **Study** | **Country** | **City** | **Assessment Method** | **sample Size** | **Age Range** | **Asthma Prevalence** | **Lower Limit** | **Upper Limit** |
| --- | --- | --- | --- | --- | --- | --- | --- | --- |
| **Strannegard, 1990** | Kuwait | - | clinical examination | 50300 | 1-12 | 12.04 | 11.76 | 12.33 |
| **Bener, 1994** | United Arab Emirates | Al-Ai | clinical examination | 9000 | 6-14 | 13.60 | 12.90 | 14.33 |
| **Abuekteish, 1996** | Jordan | Irbid | self-report | 3540 | 6-12 | 3.73 | 3.13 | 4.41 |
| **Hijazi, 1998** | Saudi Arabia | - | self-report | 1444 | 12 | 12.12 | 10.48 | 13.91 |
| **Al-Dawood, 2000** | Saudi Arabia | Al-Khobar | self-report | 1482 | 6-15 | 9.51 | 8.07 | 11.12 |
| **Al-Maskari, 2000** | United Arab Emirates | - | self-report | 3002 | 6-12 | 12.52 | 11.36 | 13.76 |
| **Alshehri, 2000** | Saudi Arabia | Abha | self-report | 4300 | 7-15 | 9.00 | 8.16 | 9.90 |
| **Behbehani, 2000** | Kuwait | Urban Kuwait | self-report | 3110 | 13-14 | 13.60 | 12.42 | 14.86 |
| **Hasan, 2000** | Palestine | Ramallah and Anin | self-report | 894 | 6-14 | 3.58 | 2.46 | 5.02 |
| **Al Frayh, 2001** | Saudi Arabia | Jeddah, Riyadh,  Hail and Gizan | self-report | 3131 | 8-16 | 12.58 | 11.44 | 13.80 |
| **El-Sharif, 2002** | Palestine | Ramallah district | self-report | 3382 | 6-12 | 9.40 | 8.44 | 10.44 |
| **Habibi-Khorasani, 2002** | Iran | Kerman | self-report | 2217 | 6-12 | 2.71 | 2.07 | 3.47 |
| **Al-Riyami, 2003** | Oman | Sultanate | self-report | 7067 | 6-14 | 15.07 | 14.24 | 15.93 |
| **El-Sharif, 2003** | Palestine | - | self-report | 14355 | 5-15 | 7.18 | 6.76 | 7.61 |
| **Al Frayh, 2005** | Saudi Arabia | - | clinical examination | 1678 | 2-6 | 21.45 | 19.51 | 23.50 |
| **Al-Thamiri, 2005** | Iraq | Baghdad | self-report | 2889 | 6-15 | 22.29 | 20.79 | 23.85 |
| **Hussein, 2005** | Sudan | Gezira | self-report | 3862 | 6-15 | 9.19 | 8.30 | 10.15 |
| **Bouayad, 2006** | Morocco | Boulmane,  Ben Slimane,  Marrakech  Casablanca | self-report | 5665 | 13-14 | 11.14 | 10.33 | 11.99 |
| **Janahi, 2006** | Qatar | - | self-report | 3283 | 6-14 | 19.77 | 18.42 | 21.17 |
| **Waked, 2006** | Lebanon | Bekaa,Beirut | self-report | 1613 | - | 5.64 | 4.57 | 6.88 |
| **Bazazi, 2007** | Iran | Gorgan | self-report | 2800 | 12-13 | 7.00 | 6.08 | 8.01 |
| **Rahimi, 2007** | Iran | Urmia | self-report | 3000 | 13-14 | 2.07 | 1.59 | 2.64 |
| **Al-Rawas, 2008** | Oman | - | self-report | 14703 | 6-14 | 15.26 | 14.68 | 15.85 |
| **Mustafa, 2008** | Pakistan | South Punjab  region | self-report | 3180 | - | 5.57 | 4.79 | 6.42 |
| **Najafizadeh, 2008** | Iran | Rasht | self-report | 6074 | 6-14 | 5.75 | 5.17 | 6.36 |
| **Owayed, 2008** | Kuwait | - | self-report | 2882 | 13-14 | 15.54 | 14.24 | 16.92 |
| **Waked, 2008** | Lebanon | - | self-report | 3909 | 5-12 | 4.86 | 4.21 | 5.58 |
| **Alsowaidi, 2009** | United Arab Emirates | - | self-report | 2802 | 13-19 | 15.49 | 14.17 | 16.88 |
| **Abu-Ekteish, 2009** | Jordan | Almafraq,Amman | self-report | 9108 | 6-14 | 13.14 | 12.45 | 13.85 |
| **Alsamarai, 2009** | Iraq | Samara | self-report | 2875 | 11-14 | 7.23 | 6.31 | 8.24 |
| **Hasnain, 2009** | Pakistan | karachi | self-report | 2325 | 3-16 | 15.78 | 14.33 | 17.33 |
| **Musharrafieh, 2009** | Lebanon | - | self-report | 3115 | 13-14 | 8.31 | 7.37 | 9.34 |
| **Ftouh, 2009** | Tunisia, Morocco | - | self-report |  | <16 | 3.55  4.38 | 2.90  3.54 | 3.45  5.35 |
| **Zedan, 2009** | Egypt | Nile Delta region | self-report | 2544 | 6-12 | 6.09 | 5.19 | 7.09 |
| **Mohammad, 2010** | Syria | - | self-report | 14175 | 6-14 | 4.79 | 4.44 | 5.15 |
| **Shakurnia, 2010** | Iran | Ahvaz | self-report | 2860 | 7-13 | 7.52 | 6.58 | 8.55 |
| **Zobeiri, 2011** | Iran | Kermanshah | self-report | 6236 | 6-13 | 2.60 | 2.22 | 3.02 |
| **Kiadeh, 2013** | Iran | Birjand | self-report | 3070 | 6-7 | 2.12 | 1.64 | 2.69 |
| **Abdallah, 2012** | Egypt | assuit | self-report | 1048 | 13-17 | 6.20 | 4.82 | 7.84 |
| **Bahaj, 2012** | Yemen | Sana’a | self-report | 1028 | 13-14 | 14.40 | 12.31 | 16.69 |
| **Ghaffari, 2012** | Iran | Sari | self-report | 1818 | 7-12 | 8.75 | 7.49 | 10.14 |
| **Ghobain, 2012** | Saudi Arabia | Riyadh | self-report | 3073 | 16-18 | 19.62 | 18.23 | 21.07 |
| **Nahhas, 2012** | Saudi Arabia | Madinah | self-report | 5188 | 6-8 | 15.48 | 14.50 | 16.49 |
| **El Sherbini, 2013** | Egypt | Fayoum | self-report | 1646 | 6-12 | 6.26 | 5.14 | 7.54 |
| **Elmoneim, 2013** | Egypt | Sohag | clinical examination | 12612 | 3-12 | 1.41 | 1.21 | 1.63 |
| **Mallol, 2013** | International | Entire EMRO | self-report | 92278 | 6-14 | 9.35 | 9.16 | 9.54 |
| **Al-Sheyab, 2014** | Jordan | northern parts | self-report | 815 | 12-13 | 11.90 | 9.76 | 14.33 |
| **Al-Sindi, 2014** | Bahrain | Not reported | self-report | 1951 | 6-7 | 11.02 | 9.66 | 12.49 |
| **Farrokhi, 2014** | Iran | Bushehr | self-report | 2395 | 6-14 | 7.01 | 6.02 | 8.11 |
| **Kalmarzi, 2016** | Iran | Kurdistan Province | self-report | 3890 | 6-14 | 3.91 | 3.32 | 4.56 |
| **Khan, 2014** | Pakistan | Karachi | self-report | 1046 | 3-17 | 10.42 | 8.63 | 12.43 |
| **Mansour, 2014** | Egypt | Damietta | self-report | 1426 | >14 | 9.12 | 7.67 | 10.73 |
| **AlBehandy, 2015** | United Arab Emirates | Dubai | self-report | 1639 | 13-19 | 16.66 | 14.88 | 18.55 |
| **Bemanin, 2015** | Afghanistan | Kabul | self-report | 3000 | 6-14 | 14.90 | 13.64 | 16.23 |
| **Moghaddam, 2015** | Iran | Not reported | self-report | 3540 | - | 2.32 | 1.85 | 2.87 |
| **Alqahtani, 2016** | Saudi Arabia | Najran | self-report | 1700 | 7-19 | 17.82 | 16.03 | 19.73 |
| **El-Mashad, 2016** | Egypt | Menoufia | self-report | 2544 | 6-12 | 6.09 | 5.19 | 7.09 |
| **Eldin, 2016** | Egypt | Fayoum | self-report | 1656 | 6-12 | 6.22 | 5.10 | 7.49 |
| **Meatty, 2016** | Egypt | Dakahlia | self-report | 1500 | 2-17 | 13.40 | 11.72 | 15.23 |
| **Mehravar, 2016** | Iran | Golestan | self-report | 1706 | 6-14 | 16.65 | 14.91 | 18.50 |
| **Ahmadiafshar, 2017** | Iran | Zanjan | self-report | 800 | 6-7 | 1.00 | 0.43 | 1.96 |
| **Al-Kubaisy, 2017** | Iraq | Baghdad | self-report | 2262 |  | 28.47 | 26.62 | 30.38 |
| **Ghanbari, 2017** | Iran | Tabriz | self-report | 1143 | 15-18 | 12.42 | 10.57 | 14.48 |
| **Khawaji, 2017** | Saudi Arabia | Jazan Region | self-report | 1400 | 10-15 | 14.71 | 12.90 | 16.68 |
| **Alruwaili, 2018** | Saudi Arabia | Arar | self-report | 511 | 16-18 | 11.35 | 8.73 | 14.42 |
| **Fareed, 2018** | Saudi Arabia | Riyadh | self-report | 1496 | ≤18 | 8.62 | 7.25 | 10.16 |
| **Halay, 2018** | Sudan | Gazera,  White Nile,  the Northern States | self-report | 3352 | 1-6 | 6.95 | 6.11 | 7.87 |
| **Mohammed, 2020** | Egypt | Elmaraghah | self-report | 1796 | 6-12 | 12.53 | 11.03 | 14.15 |
| **Sonia, 2018** | Tunisia | Tunis | self-report | 1661 | 5-15 | 5.72 | 4.65 | 6.95 |
| **al-Mutairi, 2018** | Saudi Arabia | Buraydah | self-report | 309 | 5-15 | 29.13 | 24.12 | 34.54 |
| **Al-Motlaq, 2019** | Jordan | Zarqa | self-report | 2821 | 6-18 | 5.85 | 5.01 | 6.78 |
| **Boshra, 2019** | Egypt | Assiut | self-report | 1100 | 9-12 | 7.64 | 6.14 | 9.37 |
| **Fazlollahi, 2019** | Iran | National study | self-report | 33260 | 6-14 | 4.32 | 4.11 | 4.55 |
| **Ibrahim, 2021** | United Arab Emirates | - | self-report | 3419 | 6-14 | 9.48 | 8.52 | 10.51 |
| **Veettil, 2019** | Qatar |  | clinical examination | 54704 | 5-12 | 22.05 | 21.71 | 22.40 |
| **Zahid, 2019** | Pakistan | Karachi | self-report | 544 | 12-15 | 20.04 | 16.75 | 23.65 |
| **Abdelmotaleb, 2020** | Egypt |  | self-report | 1450 | 2-16 | 15.45 | 13.63 | 17.41 |
| **Al-sheyab, 2020** | Jordan | northern parts | self-report | 2690 | 13-14 | 12.49 | 11.26 | 13.80 |
| **Behniafard, 2020** | Iran | Yazd | self-report | 5141 | 13-14 | 4.80 | 4.24 | 5.43 |
| **Sobieh, 2020** | Egypt | Qalubyia | self-report | 1000 | 2-6 | 16.40 | 14.16 | 18.84 |
| **Tavakol, 2020** | Iran | Karaj | self-report | 950 | 13_14 | 5.58 | 4.21 | 7.23 |
| **Ali, 2021** | Libya | Albadya | self-report | 478 | 6-15 | 12.55 | 9.72 | 15.86 |
| **Dastoorpoor, 2022** | Iran | Khuzestan Province | self-report | 7344 | 6-14 | 2.59 | 2.24 | 2.98 |
| **Hallit, 2021** | Lebanon | - | self-report | 515 | 3-5 | 8.16 | 5.94 | 10.86 |
| **Jamil, 2021** | United Arab Emirates | Ajman | self-report | 455 | 15-18 | 9.67 | 7.11 | 12.76 |
| **Sabeti, 2021** | Iran | Tabriz,Hadishahr | self-report | 1459 | 14-19 | 4.93 | 3.88 | 6.17 |
| **Samoo, 2021** | Pakistan | Karachi | self-report | 341 | 4-15 | 14.08 | 10.56 | 18.23 |
| **Alsaadi, 2022** | Iraq | Erbil | self-report | 2000 | 12-19 | 7.00 | 5.92 | 8.21 |
| **Alomary, 2022** | Saudi Arabia | Not reported | self-report | 7700 | 6-14 | 14.84 | 14.06 | 15.66 |
| **Alshammrie, 2022** | Saudi Arabia | Hail City | self-report | 964 | 2-8 | 18.78 | 16.36 | 21.39 |
| **Hammoudeh, 2022** | Qatar | - | self-report | 2646 | 6-14 | 8.24 | 7.22 | 9.35 |
| **Alatawi, 2023** | Saudi Arabia | Tabuk | self-report | 384 | 5-19 | 31.77 | 27.14 | 36.69 |
| **Mohammad, 2023** | Syria | Latakia,Damascus | self-report | 2315 | 13–14 | 12.83 | 11.49 | 14.26 |
| **Nour, 2023** | Jordan | Amman | self-report | 5319 | - | 2.54 | 2.13 | 3.00 |
| **Gohal, 2024** | Saudi Arabia | Jazan | clinical examination | 1368 | 5-18 | 24.49 | 22.23 | 26.86 |

**Table B2: Risk of bias assessment of included studies using Joanna Briggs Institute tool.**

| **Author** | **Year** | **Q1** | **Q2** | **Q3** | **Q4** | **Q5** | **Q6** | **Q7** | **Q8** | **Q9** | **JBI Score** |
| --- | --- | --- | --- | --- | --- | --- | --- | --- | --- | --- | --- |
| Strannegard | 1990 | Yes | Yes | Yes | Yes | No | Yes | Yes | Unclear | No | 0.72 |
| Bener | 1994 | Yes | Yes | Yes | Yes | Unclear | Yes | Yes | Unclear | No | 0.78 |
| Abuekteish | 1996 | Yes | Yes | Yes | Yes | Yes | Yes | Yes | Yes | Yes | 1.00 |
| Hijazi | 1998 | Yes | Yes | Yes | Yes | Yes | Yes | Yes | Unclear | Unclear | 0.89 |
| Al-Dawood | 2000 | Yes | Yes | Yes | Yes | Yes | Yes | Yes | Unclear | Unclear | 0.89 |
| Al-Maskari | 2000 | Yes | Yes | Yes | Yes | Yes | Yes | Yes | Yes | Yes | 1.00 |
| Alshehri | 2000 | Yes | Yes | Yes | Yes | Yes | Yes | Yes | Unclear | Yes | 0.94 |
| Behbehani | 2000 | Yes | Yes | Yes | Yes | Yes | Yes | Yes | Unclear | Yes | 0.94 |
| Hasan | 2000 | Yes | Yes | Yes | Yes | Yes | Yes | Yes | Yes | Yes | 1.00 |
| Al Frayh | 2001 | Yes | Yes | Yes | Yes | Yes | Yes | Yes | Unclear | Unclear | 0.89 |
| El-Sharif | 2002 | Yes | Yes | Yes | Yes | Yes | Yes | Yes | Yes | Yes | 1.00 |
| Habibi-Khorasani | 2002 | Yes | Yes | Yes | Yes | No | Yes | Yes | Yes | No | 0.78 |
| Al-Riyami | 2003 | Yes | Yes | Yes | Yes | Yes | Yes | Yes | Unclear | No | 0.83 |
| El-Sharif | 2003 | Yes | Yes | Yes | Yes | Yes | Yes | Yes | Unclear | Unclear | 0.89 |
| Al Frayh | 2005 | Yes | Yes | Yes | Yes | Yes | Yes | Yes | Yes | Yes | 1.00 |
| Al-Thamiri | 2005 | Yes | Yes | Yes | Yes | No | Yes | Yes | Yes | No | 0.78 |
| Hussein | 2005 | Yes | Yes | Yes | Yes | Yes | Yes | Yes | Unclear | Yes | 0.94 |
| Bouayad | 2006 | Yes | Yes | Yes | Yes | Yes | Yes | Yes | Yes | Yes | 1.00 |
| Janahi | 2006 | Yes | Yes | Yes | Yes | Yes | Yes | Yes | Yes | Yes | 1.00 |
| Waked | 2006 | Yes | Yes | Yes | Yes | Yes | Yes | Yes | Yes | Yes | 1.00 |
| Bazazi | 2007 | Yes | No | No | Yes | Unclear | Yes | Yes | Yes | Yes | 0.72 |
| Rahimi | 2007 | Yes | Unclear | Yes | Yes | Yes | Yes | Yes | Yes | Yes | 0.94 |
| Al-Rawas | 2008 | Yes | Yes | Yes | Yes | Yes | Yes | Yes | Yes | Yes | 1.00 |
| Mustafa | 2008 | Yes | Yes | Yes | Yes | Yes | Yes | Yes | Yes | Yes | 1.00 |
| Najafizadeh | 2008 | Yes | Yes | Yes | Yes | Yes | Yes | Yes | Yes | Yes | 1.00 |
| Owayed | 2008 | Yes | Yes | Yes | Yes | Yes | Yes | Yes | Yes | Yes | 1.00 |
| Waked | 2008 | Yes | Yes | Yes | Yes | Yes | Yes | Yes | Yes | Yes | 1.00 |
| Alsowaidi | 2009 | Yes | Yes | Yes | Yes | Yes | Yes | Yes | Yes | Yes | 1.00 |
| Abu-Ekteish | 2009 | Yes | Yes | Yes | Yes | Yes | Yes | Yes | Yes | Yes | 1.00 |
| Alsamarai | 2009 | Yes | Yes | Yes | Yes | Yes | Yes | Yes | Yes | Yes | 1.00 |
| Ftouh | 2009 | Yes | Yes | Unclear | No | Unclear | Yes | Yes | No | Yes | 0.67 |
| Hasnain | 2009 | Yes | Yes | Yes | Yes | Yes | Yes | Yes | Yes | Yes | 1.00 |
| Musharrafieh | 2009 | Yes | Yes | Yes | Yes | Yes | Yes | Yes | Yes | Yes | 1.00 |
| Zedan | 2009 | Yes | Yes | Yes | Yes | Yes | Yes | Yes | Yes | Yes | 1.00 |
| Mohammad | 2010 | Yes | Yes | Yes | Yes | Yes | Yes | Yes | Yes | Yes | 1.00 |
| Shakurnia | 2010 | Yes | Yes | Yes | Yes | Yes | Yes | Yes | Yes | Yes | 1.00 |
| Zobeiri | 2011 | Yes | Yes | Yes | Yes | Yes | Yes | Yes | Yes | Yes | 1.00 |
| Kiadeh | 2013 | Yes | Yes | Yes | Yes | Yes | Yes | Yes | Yes | Yes | 1.00 |
| Abdallah | 2012 | Yes | Yes | Yes | Yes | Yes | Yes | Yes | Yes | Yes | 1.00 |
| Bahaj | 2012 | Yes | Yes | Yes | Yes | Yes | Yes | Yes | Yes | Yes | 1.00 |
| Ghaffari | 2012 | Yes | Yes | Yes | Yes | Yes | Yes | Yes | Yes | Yes | 1.00 |
| Ghobain | 2012 | Yes | Yes | Yes | Yes | Yes | Yes | Yes | Yes | Yes | 1.00 |
| Nahhas | 2012 | Yes | Yes | Yes | Yes | Yes | Yes | Yes | Yes | Yes | 1.00 |
| El Sherbini | 2013 | Yes | Yes | Yes | Yes | Yes | Yes | Yes | Yes | Yes | 1.00 |
| Elmoneim | 2013 | Yes | Yes | Yes | Yes | Yes | Yes | Yes | Yes | Unclear | 0.94 |
| Mallol | 2013 | Yes | Yes | Yes | Yes | Yes | Yes | Yes | Yes | Yes | 1.00 |
| Al-Sheyab | 2014 | No | Unclear | No | Unclear | Unclear | Yes | Yes | Yes | Unclear | 0.56 |
| Al-Sindi | 2014 | Yes | Yes | Yes | Yes | No | Yes | Yes | Yes | Unclear | 0.83 |
| Farrokhi | 2014 | No | Yes | Yes | Yes | Yes | Yes | Yes | Yes | Yes | 0.89 |
| Kalmarzi | 2016 | Yes | Yes | Yes | Yes | Yes | Yes | Yes | Unclear | Unclear | 0.89 |
| Khan | 2014 | Yes | Yes | Yes | Yes | No | Yes | Yes | Yes | Unclear | 0.83 |
| Mansour | 2014 | Yes | Yes | Yes | Yes | Yes | Yes | Yes | Yes | Yes | 1.00 |
| AlBehandy | 2015 | Yes | Yes | Yes | Yes | Yes | Yes | Yes | Yes | Yes | 1.00 |
| Bemanin | 2015 | Yes | Yes | Yes | Yes | Yes | Yes | Yes | Yes | Yes | 1.00 |
| Moghaddam | 2015 | Yes | Yes | Yes | Yes | Yes | Yes | Yes | Yes | Yes | 1.00 |
| Alqahtani | 2016 | Yes | Yes | Yes | Yes | Yes | Yes | Yes | Yes | Yes | 1.00 |
| El-Mashad | 2016 | Yes | Yes | Yes | Yes | Unclear | Yes | Yes | Yes | Unclear | 0.89 |
| Eldin | 2016 | Yes | Yes | Yes | Yes | Yes | Yes | Yes | Yes | Yes | 1.00 |
| Meatty | 2016 | Yes | Yes | Yes | Yes | Yes | Yes | Yes | Yes | Yes | 1.00 |
| Mehravar | 2016 | Unclear | Yes | Yes | Yes | Yes | Yes | Yes | Yes | No | 0.83 |
| Ahmadiafshar | 2017 | Yes | Yes | Yes | Yes | Yes | Yes | Yes | Yes | Yes | 1.00 |
| Al-Kubaisy | 2017 | Yes | Yes | Unclear | Yes | Unclear | Yes | Yes | Yes | Yes | 0.89 |
| Ghanbari | 2017 | Yes | Yes | Yes | Yes | Yes | Yes | Yes | Unclear | Yes | 0.94 |
| Khawaji | 2017 | Yes | Yes | Yes | Yes | Yes | Yes | Yes | Yes | Yes | 1.00 |
| Alruwaili | 2018 | Unclear | Unclear | Unclear | Yes | No | Yes | Yes | Unclear | Yes | 0.67 |
| Fareed | 2018 | Yes | Yes | Yes | Yes | Yes | Yes | Yes | Yes | Yes | 1.00 |
| Halay | 2018 | Yes | Yes | Yes | Yes | Yes | Yes | Yes | Yes | Yes | 1.00 |
| Mohammed | 2020 | Yes | Yes | Yes | Yes | Yes | Yes | Yes | Yes | Yes | 1.00 |
| Sonia | 2018 | Yes | Yes | Yes | Yes | Yes | Yes | Yes | Yes | Yes | 1.00 |
| al-Mutairi | 2018 | Yes | Yes | No | Yes | Yes | Yes | Yes | Unclear | Unclear | 0.78 |
| Al-Motlaq | 2019 | Yes | Yes | Yes | Yes | Yes | Yes | Yes | Yes | Yes | 1.00 |
| Boshra | 2019 | Yes | Yes | Yes | Yes | Yes | Yes | Yes | Yes | Yes | 1.00 |
| Fazlollahi | 2019 | Yes | Yes | Yes | Yes | Yes | Yes | Yes | Yes | Yes | 1.00 |
| Ibrahim | 2021 | Yes | Yes | Yes | Yes | Yes | Yes | Yes | Yes | Yes | 1.00 |
| Veettil | 2019 | Yes | Yes | Yes | Yes | Yes | Yes | Yes | Yes | Yes | 1.00 |
| Zahid | 2019 | Unclear | Yes | Unclear | Unclear | Unclear | Yes | Yes | Unclear | Unclear | 0.67 |
| Abdelmotaleb | 2020 | Yes | Yes | Yes | Yes | Yes | Yes | Yes | Yes | Yes | 1.00 |
| Al-sheyab | 2020 | Yes | Yes | Yes | Yes | Yes | Yes | Yes | Yes | Yes | 1.00 |
| Behniafard | 2020 | Yes | Yes | Yes | Yes | Yes | Yes | Yes | Yes | Yes | 1.00 |
| Sobieh | 2020 | Yes | Yes | Yes | Yes | Yes | Yes | Yes | Yes | Yes | 1.00 |
| Tavakol | 2020 | Yes | Yes | Yes | Yes | Yes | Yes | Yes | Yes | Yes | 1.00 |
| Ali | 2021 | No | Yes | Yes | Yes | Yes | Yes | Yes | No | Yes | 0.78 |
| Dastoorpoor | 2022 | Yes | Yes | Yes | Yes | Yes | Yes | Yes | Yes | Yes | 1.00 |
| Hallit | 2021 | Unclear | Yes | Unclear | Unclear | Unclear | Yes | Yes | Unclear | Yes | 0.72 |
| Jamil | 2021 | Yes | Unclear | No | Unclear | Unclear | Yes | Yes | Unclear | Yes | 0.67 |
| Sabeti | 2021 | Yes | Yes | Yes | Yes | Yes | Yes | Yes | Yes | Yes | 1.00 |
| Samoo | 2021 | Yes | Yes | No | Yes | Yes | Yes | Yes | Unclear | Unclear | 0.78 |
| Alsaadi | 2022 | Yes | Yes | Unclear | No | Unclear | Yes | Yes | No | Yes | 0.67 |
| Alomary | 2022 | Yes | Yes | Yes | Yes | Yes | Yes | Yes | Yes | Yes | 1.00 |
| Alshammrie | 2022 | Yes | Yes | Yes | Yes | Yes | Yes | Yes | Yes | Yes | 1.00 |
| Hammoudeh | 2022 | Yes | Yes | Yes | Yes | Yes | Yes | Yes | Yes | Yes | 1.00 |
| Alatawi | 2023 | Yes | Yes | No | Yes | Yes | Yes | Yes | Unclear | Yes | 0.83 |
| Mohammad | 2023 | Yes | Yes | No | Yes | Yes | Yes | Yes | Yes | Yes | 0.89 |
| Nour | 2023 | Yes | Yes | Yes | Yes | Yes | Yes | Yes | Yes | Yes | 1.00 |
| Gohal | 2024 | Yes | Yes | Yes | Yes | Yes | Yes | Yes | Yes | Yes | 1.00 |

**Appendix C: Forst plots for subgroup analyses**

Figure C1: Forest plots for the pooled prevalence of asthma among girls


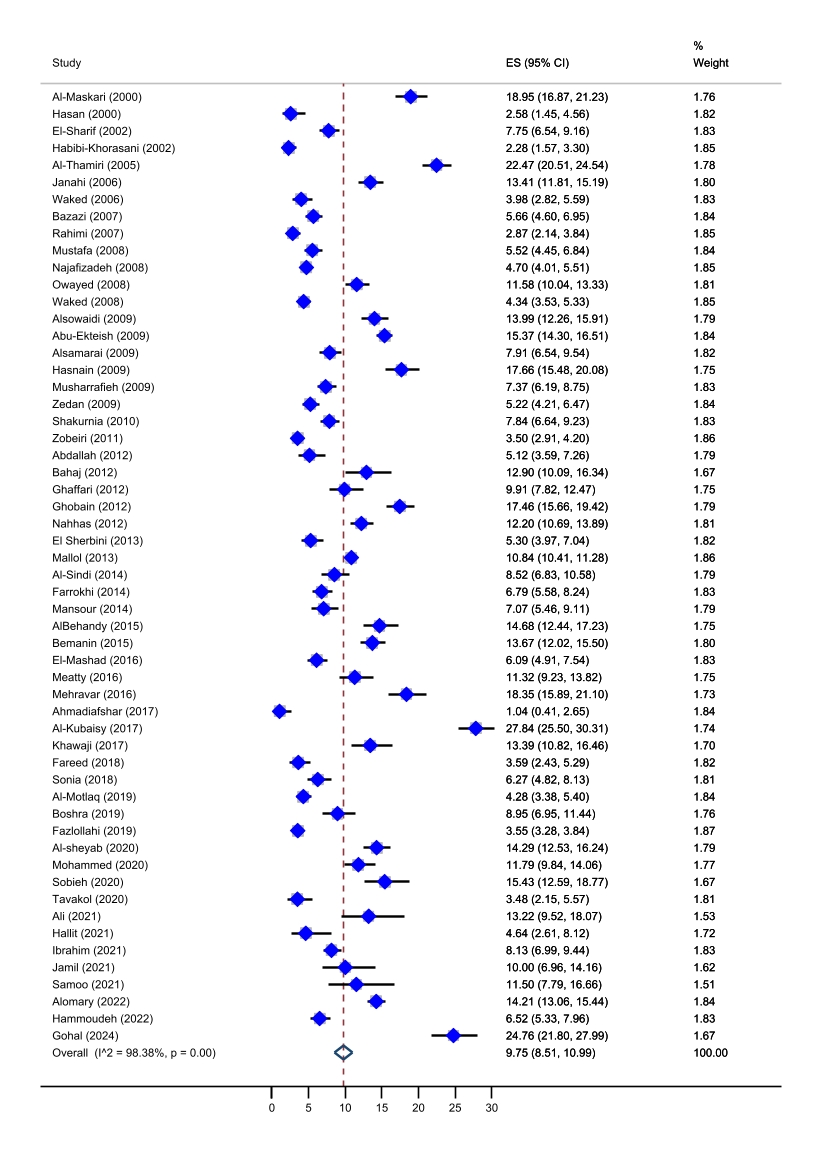


Figure C2: Forest plots for the pooled prevalence of asthma among boys


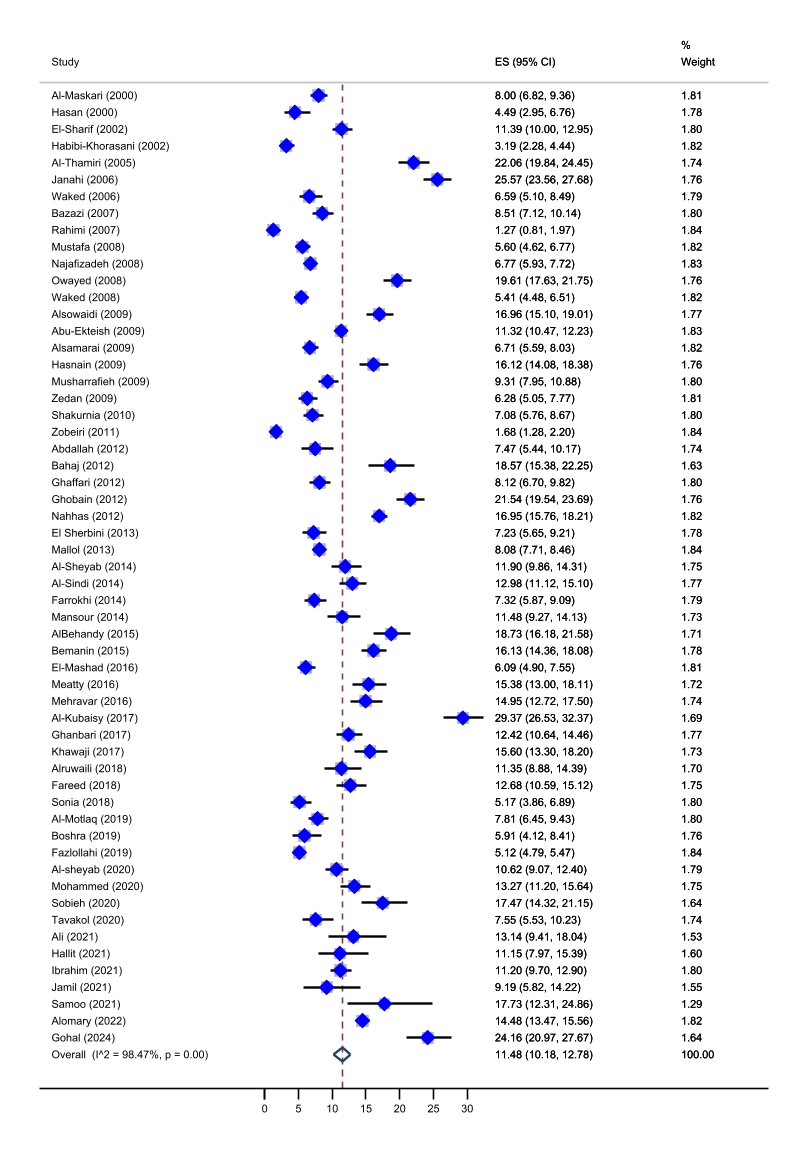


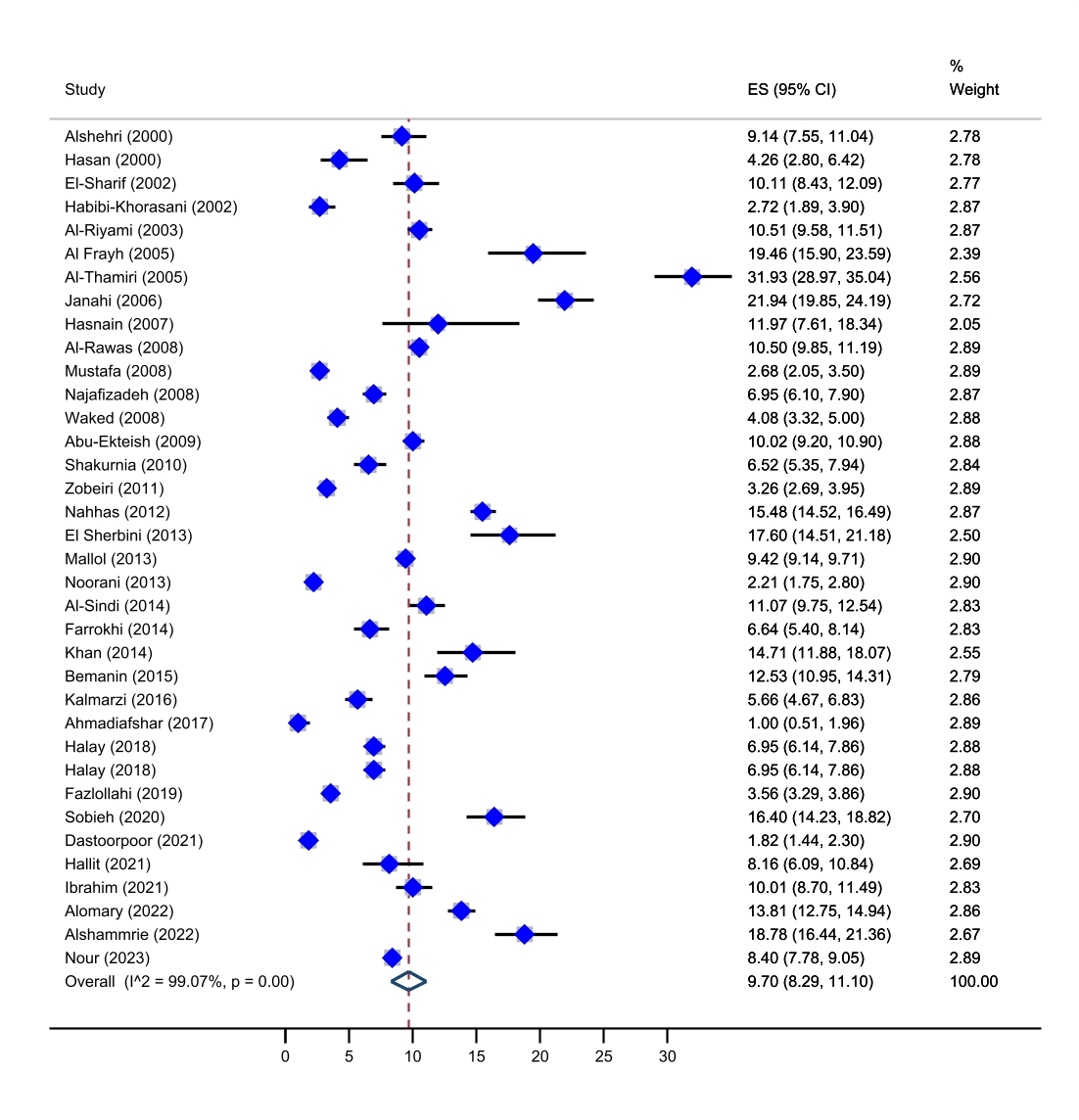


Figure C3: Forest plots for the pooled prevalence of asthma among children (under 10 years old)


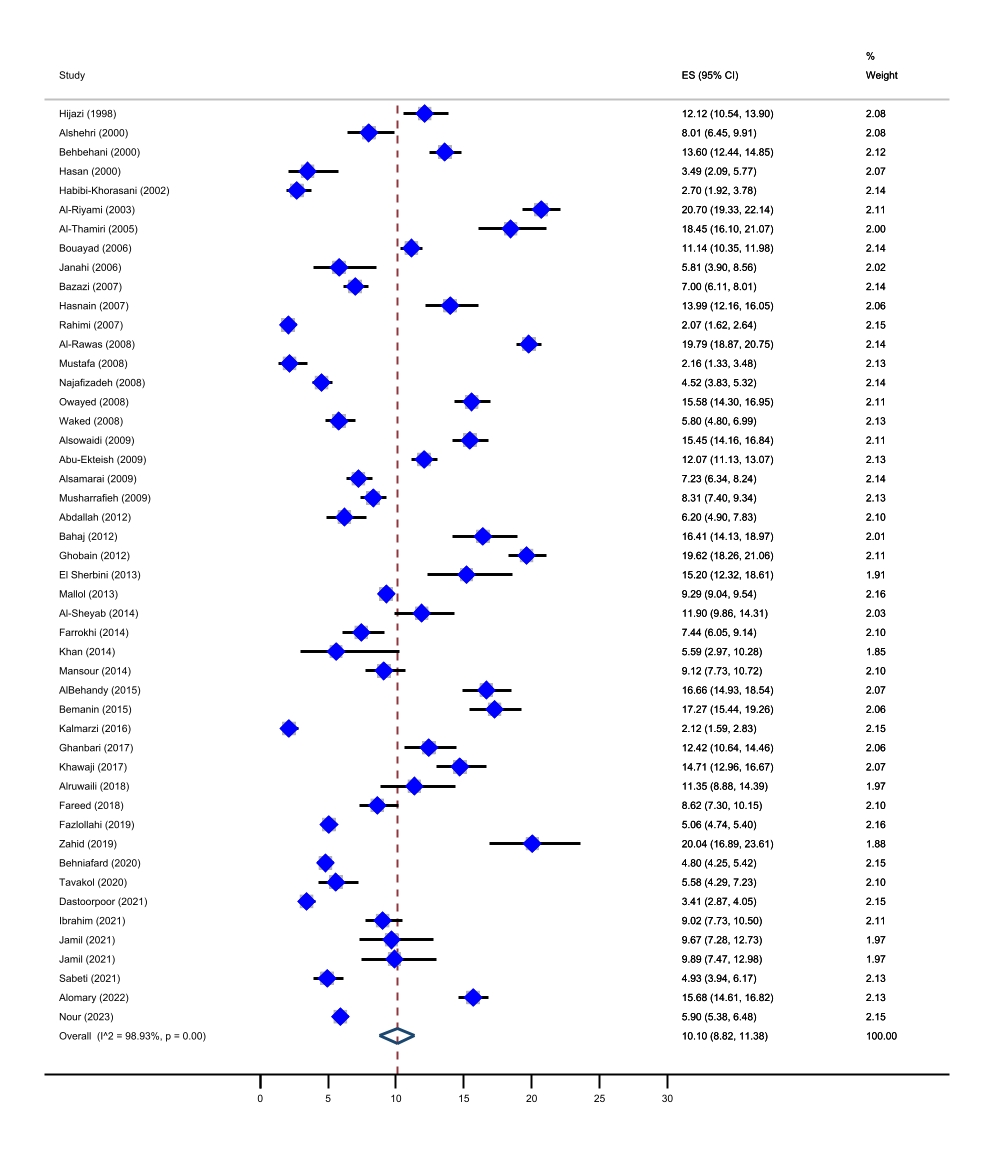


Figure C4: Forest plots for the pooled prevalence of asthma among adolescents


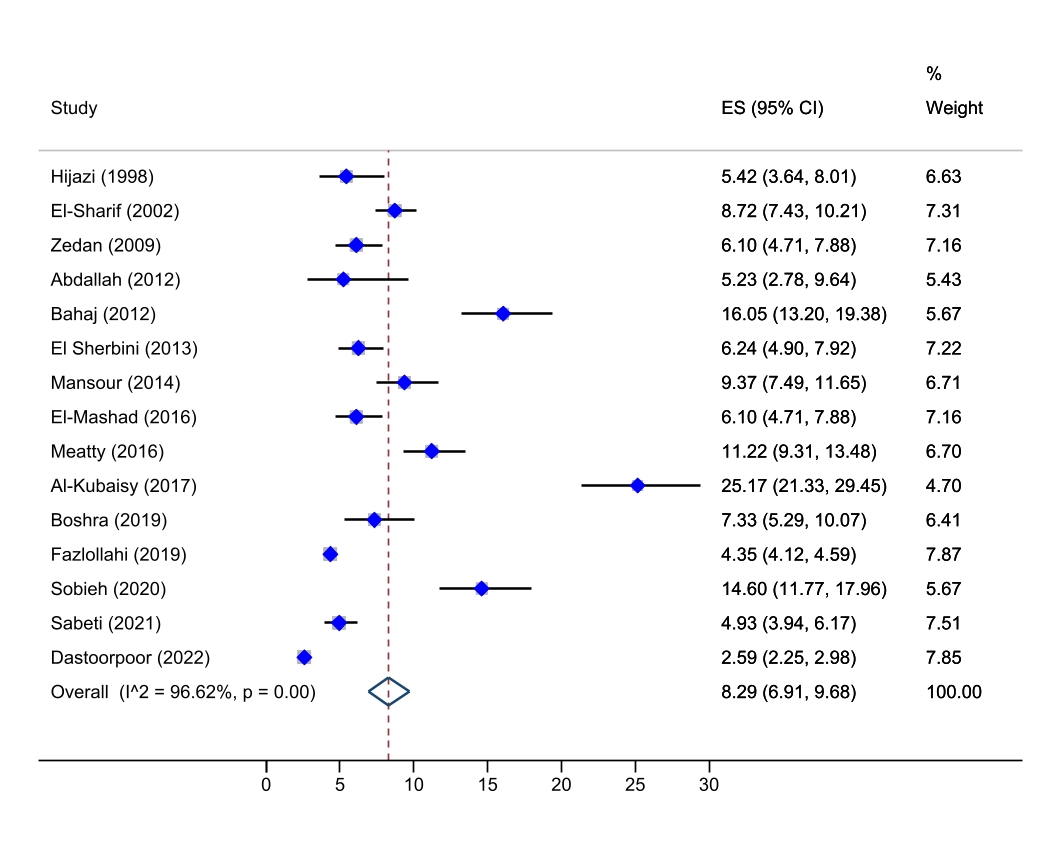


Figure C5: Forest plots for the pooled prevalence of asthma in rural area


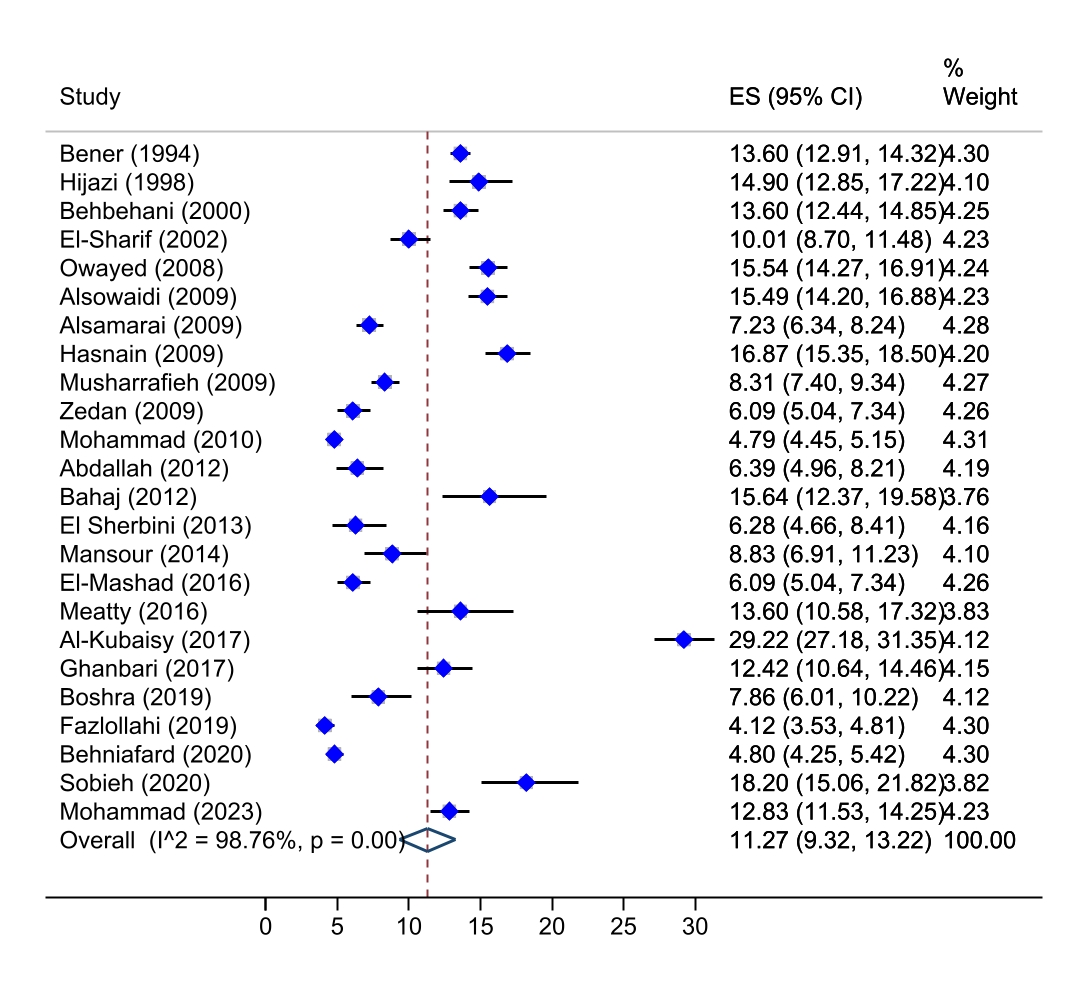


Figure C6: Forest plots for the pooled prevalence of asthma in urban area


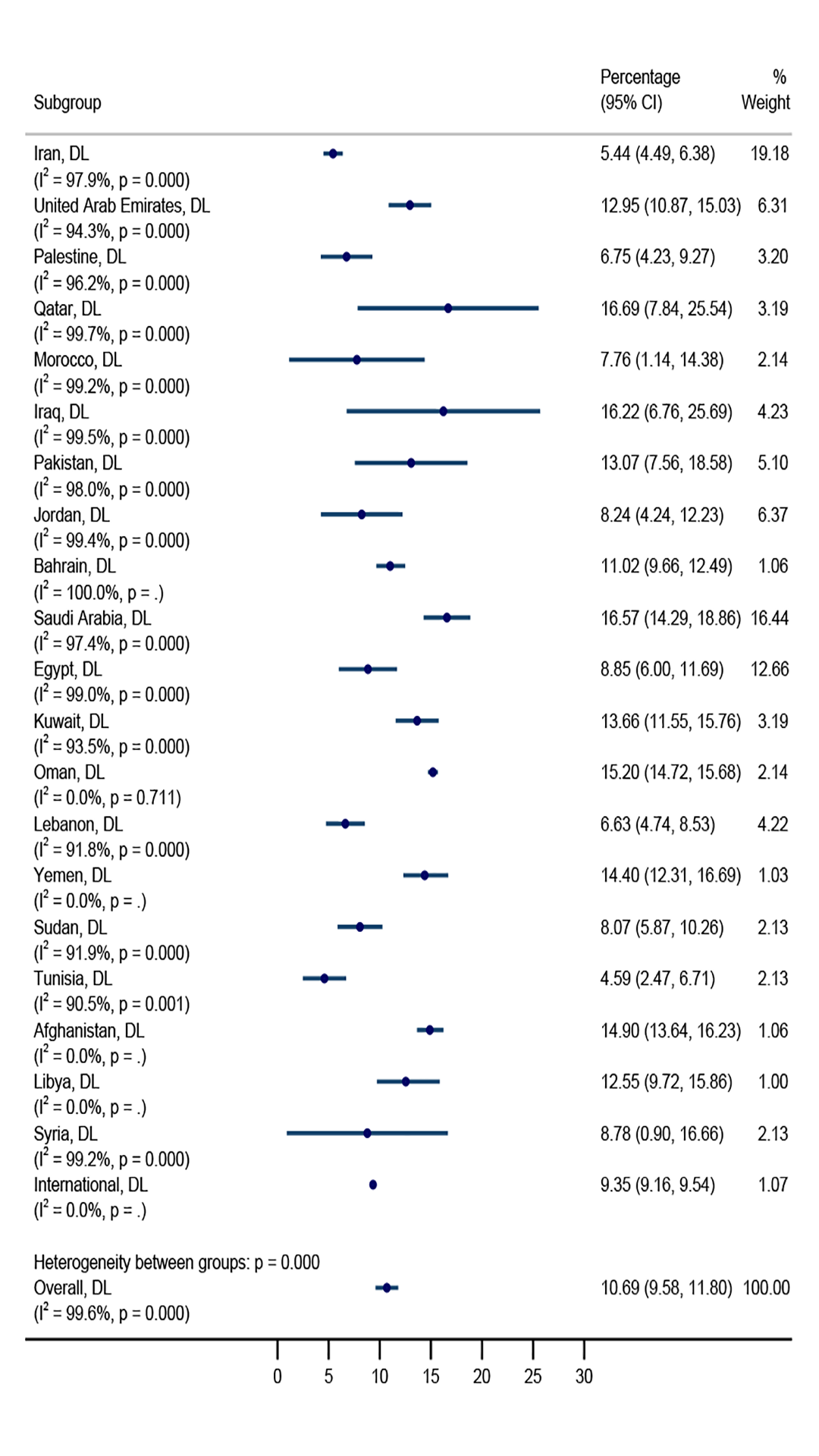


Figure C7: Forest plots depicting sensitivity analysis, excluding non-English (Persian) studies, to evaluate language influence and validate main analysis conclusions
